# GARN3: A coarse-grained helix centered technique for RNA 3D structures prediction

**DOI:** 10.1371/journal.pone.0328609

**Published:** 2026-06-22

**Authors:** Jhonatan Silva, Johanne Cohen, Daniel Cordeiro

**Affiliations:** 1 Escola de Artes, Ciências e Humanidades, Universidade de São Paulo, São Paulo, São Paulo, Brazil; 2 LISN CNRS-INRIA, Université Paris-Saclay, Gif-sur-Yvette, France; National University of Singapore, SINGAPORE

## Abstract

The study of predicting three-dimensional structures of RNA (ribonucleic acids) has increased over the last few decades, especially with advances in artificial intelligence. Despite these advances, there are still many gaps. Among the known techniques, the GARN (Game Algorithms for RNa 3D sampling) framework has demonstrated good performance on large RNA molecules. Nevertheless, the GARN technique also left room for improvement in the final 3D structures of predicted molecules, which can be further refined by including additional elements, also known as pseudoatoms. We present GARN3, an extension of GARN2 in which additional pseudoatoms are placed along helices to improve the granularity of 3D models, and a machine learning component is incorporated into the scoring function to estimate interaction distances. In our experiments, GARN3 achieved RMSD values comparable to or better than those of several existing RNA 3D structure prediction methods. TM-score evaluations indicate that GARN3 achieves a consistent global structural accuracy across multiple molecules, comparable to that of several existing methods. Relative to previous versions of GARN, GARN3 lowers the RMSD on most molecules in Test Set A and remains competitive on Test Set B (CASP targets), while providing a finer coarse-grained representation; performance is particularly consistent on large RNA structures. The implementation of the GARN3 technique is publicly available at https://github.com/jhonatans01/garn3, written and executable in Java.

## 1. Introduction

Ribonucleic acids (RNA) are molecules present in living beings that perform functions such as protein synthesis through the translation of the genetic code, catalyzing biochemical reactions, and regulating gene expression. Studies reinforce the importance of 3D structures of RNA, for example, in the production of certain drugs, and can also provide the discovery of disease-causing mutations [1].

To obtain such structures with the lowest possible error rate, experimental techniques are used in the laboratory; however, they are time-consuming and expensive [2]. For this reason, computational techniques have been developed over the past few decades to predict such structures. Recently, highly successful techniques, such as AlphaFold 3 [3], have used artificial intelligence to perform simulations. The techniques are currently open source and are available to researchers. However, there are limitations, such as the need for substantial computational resources and/or the length of the molecule sequence to be predicted.

Many prediction techniques benefit from experimentally determined RNA structures. These techniques are usually developed by taking advantage of physics interactions [4,5], statistical potentials such as knowledge-based (KB) functions [6–11], or even databases of published molecules to simulate structures of new molecules [12–16]. One of these studies, which uses statistical potentials, GARN (*Game Algorithms for RNa 3D sampling*) [10,17], has demonstrated the ability to overcome the time and length limitations of previous techniques by applying game-theoretic concepts in its molecular simulations. Game theory is an area of mathematics whose objective is to analyze interactions between two or more elements within a set, where the actions of one element must always be taken by checking the possible actions of the others [18].

In GARN, simulations use a coarse-grained representation based on a 3D graph. It starts with the algorithm’s input, namely the primary and secondary structures, and generates the initial 3D graph, with nodes representing the molecule’s nucleotides. Each node of the graph represents one or more nucleotides, relying on the Secondary Structure Element (SSE), such as helices, stem-loops, and *k-way* junctions. The nodes of the graph are linked using a predefined distance based on the node’s SSE type. To determine the position of each node, game theory is applied as follows: the players are the nodes of the graph, and the strategies consist of predefined angles through which one player is connected to another, aggregated by SSE type. Each game aims to generate a possible molecule by moving each player according to the selected strategy. At each turn, the probability of each strategy being chosen by each player is updated using two machine-learning-based regret-minimization algorithms. The goal of these algorithms is to maximize gain or minimize loss based on each player’s payoff.

The payoff for each player is defined by a KB function, using a well-known intermolecular potential named Lennard–Jones [19]. The inputs of this KB function were defined after several analyses of native molecules as well as the predefined strategies of the game. The simulation ends after multiple games are generated, using the concept of the multi-armed bandit problem [20], where many “arms” (strategies) are pulled to play the games, and the game that achieves the best result is chosen to build the final 3D structure.

Taking into account previous studies on the technique GARN [10,17], the main difference is that GARN2 is able to predict molecules with 4-way junctions or higher-order junctions, whereas GARN can only predict molecules with topologies up to 3-way junctions. However, previous GARN studies identified opportunities to improve the prediction of small molecules (usually fewer than 100 nucleotides), where the 3D model contains relatively few pseudoatoms compared to the number of nucleotides in the structure. A possible solution proposed by the authors is to adjust the game settings to obtain more refined structures containing more pseudoatoms, that is, visually closer to those obtained by experimental techniques.

To overcome the limitations of small-molecule predictions and improve the 3D models of all-molecule predictions, we introduce GARN3, a knowledge-based technique that predicts coarse-grained 3D structures of RNA using statistical potentials trained with machine learning. The main improvement over previous GARN studies is the updated graph structure, which adds more elements (pseudoatoms) to the final 3D structure. In previous studies, the SSE helix node aggregated several base pairs, depending on helix length, up to a maximum of 5 per node. In the updated graph, each base pair is represented by a single node, regardless of the helix length. To present the new graph with more pseudoatoms, the distances between nodes, the strategies used in the game, and the scoring function parameters were refined and updated.

Furthermore, the scoring function from the previous study considered only static parameter values. In this study, we use a machine learning model to predict distances by SSE type from the scoring function. With the new scoring function, we observe improved predictions compared to GARN2, since the machine learning model in GARN3 considers additional molecular characteristics. In addition, the scoring function can be easily updated in subsequent studies, further improving the GARN model.

## 2. Related works

To validate the GARN3 technique’s results, we compared its predictions with those of other established methods. To this end, we reviewed previous studies describing RNA 3D structure prediction techniques that use primary and/or secondary structure as input, focusing specifically on methods available online. For this purpose, recent review studies provide a mapping of techniques for predicting 3D RNA structures [21,22,23].

The techniques described in the reviews are commonly classified by the type of simulation used. The techniques FARNA [4]/ FARFAR/ FARFAR2 [12], MC-Sym [24], vFold [25]/ vFoldLA [15], RNAComposer [13], 3dRNA [26]/ 3dRNA v2 [16] and FebRNA [27] identified in the review by Clément et al. [21] are classified as *template-based* (or *fragment-assembly*), meaning that RNA experimental structures (obtained in experimental techniques) are used to derive others, either entirely or partially. The studies for the ModeRNA [28] and RNABuilder [29] techniques are also in this category and are mentioned in the reviews; however, unlike the other studies in this category, ModeRNA requires a 3D structure as input, and RNABuilder requires base-pair interactions as input, in addition to the secondary structure.

Another category is *template-free* (*ab initio*), which aggregates techniques that do not use RNA experimental structures in the predictions. These techniques generally make use of physical interactions or knowledge-based statistical potentials [30], based on observations from previous analyses of RNA experimental structures. In the review by Clément et al. [21], the techniques iFoldRNA [5], NAST [6], Ernwin [8], IsRNA [31]/ IsRNA1 [11], SimRNA [9], RNAJAG [7], RNAJP [7] are classified as template-free. Other techniques found in the review [21] are OxRNA [32], HIRE-RNA [33] and, in the review by Mukherjee et al. [22], the RNA-BRiQ [34] technique is found; however, unlike the other techniques in this category, the RNA-BRiQ and OxRNA techniques require complex inputs not found as primary and secondary structures, HIRE-RNA technique is not available online and BARNACLE [35], although its source code is publicly available, was reported as not runnable in the review by Clément et al. [21].

The last category mentioned in the reviews [21–23] is referred to as *deep learning*, which includes techniques that use artificial intelligence models that incorporate methods such as multiple sequence alignment (comparison between primary and secondary structures to find similarities and extract functions or geometric 2D/3D patterns) and geometric features (angles and distances between atoms) [23]. In the reviews [21–23], the techniques found are AlphaFold 3 [3], RhoFold + , trRosettaRNA [36], DRfold [37], RoseTTAFoldNA [38], PAMNet [39] and epRNA [40]. Another technique found in the reviews [21,22] is NuFold [41], which has become publicly available since the preparation of this study and was therefore not included in our quantitative comparison.

## 3. Materials and methods

### 3.1. Overview of the GARN3 approach

GARN3 predicts coarse-grained RNA three-dimensional structures using a game-based simulation framework. Starting from the primary and secondary structures of an RNA molecule, the method: (1) builds a three-dimensional graph representation of the molecule; (2) simulates 3D configurations using regret-minimization algorithms; (3) scores candidate configurations using a knowledge-based interaction model; (4) selects the best predicted structure according to a predefined ranking criterion.

Compared to previous versions (GARN and GARN2), GARN3 introduces a refined graph representation and a scoring function that integrates distance estimates obtained from a machine learning regression model.

The GARN3 technique uses game theory concepts. Game theory is the study of interactions among a set of elements, called players, where the actions of each player influence the outcomes of the others; the possible actions available to players in a game are called strategies [18]. Each strategy chosen by each player in a game results in a reward or a penalty for all the players. Therefore, a strategy may benefit one player more than others. To achieve an equilibrium in the rewards and penalties of all players in a game, a possible approach is called regret minimization. Regret minimization is a widely used decision-making approach for unknown environments, in which we can evaluate multiple possible game outcomes by applying a scoring function to players after choosing a possible strategy and, in the end, select strategies that provide a good balance of rewards and penalties for all players [18].

In GARN3, the players are pseudoatoms of a molecule, the strategies are the directions in which the next pseudoatom can be placed in 3D space, and the rewards (or penalties) for each player are determined by a KB scoring function applied within regret-minimization algorithms. In the context of this study, the KB function reflects the best distance between two pseudoatoms in space, considering characteristics such as SSE type and molecular length. Unlike the GARN/GARN2 [10,17] techniques, in GARN3, the best distance used in the KB function is predicted by a machine learning model rather than relying on frequency-based parameter values. This latter approach accounts for molecular characteristics such as the SSE types present in the molecule, the overall molecular length, and the length of helices, among other features described in this section.

The input to GARN simulations is the primary and secondary structure of a molecule, and the output is a file containing the 3D coordinates of that molecule. Here, the 3D coordinates refer to the coarse-grained nodes (players/pseudoatoms) of the graph representation, and not to full all-atom coordinates.

To enhance the GARN model by reducing granularity, we propose a refined 3D model that includes additional pseudoatoms. GARN configurations were updated using information derived from a dataset of 580 molecules. This dataset provides information on the primary, secondary, and 3D structures of these molecules. A detailed statistical analysis of the frequency distribution of angles and distances between atoms in molecules is presented and is used to motivate a proposal of a new set of strategies for the game, as well as new parameters for the scoring function that are better suited to the proposed representation model with reduced granularity.

This section presents the regret-minimization algorithms used in the GARN3 technique. At the end of each molecule simulation, after rounds of a game, a sorting criterion is applied to identify the structure selected as the final prediction. The sorting criteria used to rank the generated structures are also described in this section. In addition, this section explains the relationship between the players and the SSEs within the 3D representation and how the final 3D model is constructed.

### 3.2. Molecules dataset

Along with the molecules from RNA FRABASE, the test set to evaluate the predictions also includes several targets from CASP (Critical Assessment of Structure Prediction), more specifically CASP15 [42] and CASP16 [43]. For this set, we selected RNA targets with experimentally determined coordinates, also available in the RCSB Protein Data Bank. The primary sequences were obtained from the CASP public website (https://predictioncenter.org), and the secondary structures were retrieved from the NAKB database [44].

The sample consists of 580 molecules for data analysis and 42 molecules as a test set used for comparison with the predictions in this paper. The 580 molecules were exclusively used for parameter estimation and model training, while the molecules in the test set were never used during parameter tuning or model fitting. This number was determined based on the following considerations:

- Several molecules with almost identical SSEs and 3D structures were excluded to avoid bias due to repeated inclusion of highly similar entries. Similarity was quantified by computing all pairwise RMSD [45] and TM-score [46] values among the candidate molecules; whenever two molecules satisfied both the conjunctive thresholds RMSD<6.0 Å *and* TM-score>0.5, only one representative was retained in the analysis dataset, with preference given to the entry of higher resolution in the PDB.
- The same redundancy filter was applied between the analysis dataset (the 580 molecules) and the two test sets (Test Set A and Test Set B): every molecule in either test set was checked against all 580 molecules using the same RMSD/TM-score thresholds, and any candidate from the analysis dataset matching a test-set molecule was excluded from training.
- Molecules with an incomplete 3D structure were excluded because the distances and angles between the atoms in the nucleotides led to inconclusive results.

The dataset includes molecules of varying sizes and topologies, including 2-way junctions, 3-way junctions, higher-order junctions, and molecules containing pseudoknots. The list of molecules used for the analysis and for training the machine learning model is presented in S1 Appendix. To help distinguish the test sets in this work, we refer as Test Set A for the molecules with primary/secondary structures from RNA FRABASE (see S1 Table), and Test Set B for the selected molecules from CASP evaluation (see S2 Table).

### 3.3. Graph representation

We represent each RNA molecule as a three-dimensional graph derived from its secondary structure. In this graph, the vertices (nodes) correspond to structural elements (referred to as players in the game-theoretic formulation), and the edges encode the connectivity imposed by SSEs. The graph is then embedded in ${\mathbb{R}}^{3}$, and the embedding is progressively refined during the computation of a Nash equilibrium. Each vertex is associated with a specific SSE type, which determines both its connectivity and its geometric constraints. Except for the helices, the other elements in the final three-dimensional representation of the molecule follow the representation introduced in GARN [10].

**Fig 1:**
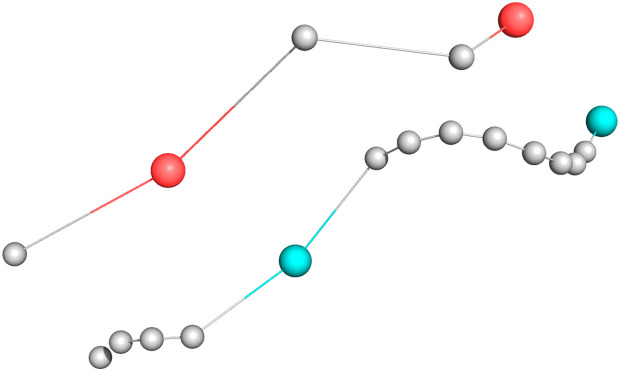
3D molecular representations using GARN-based models. The GARN/GARN2 model is shown with nodes in orange, while the GARN3 model uses cyan nodes. Both representations are generated from the native PDB structure obtained from the RCSB database (PDB ID: 1MNX). Gray nodes represent base-paired regions, whereas colored nodes indicate unpaired secondary structure elements, such as terminal loops and k-way junctions.

**Fig 2:**
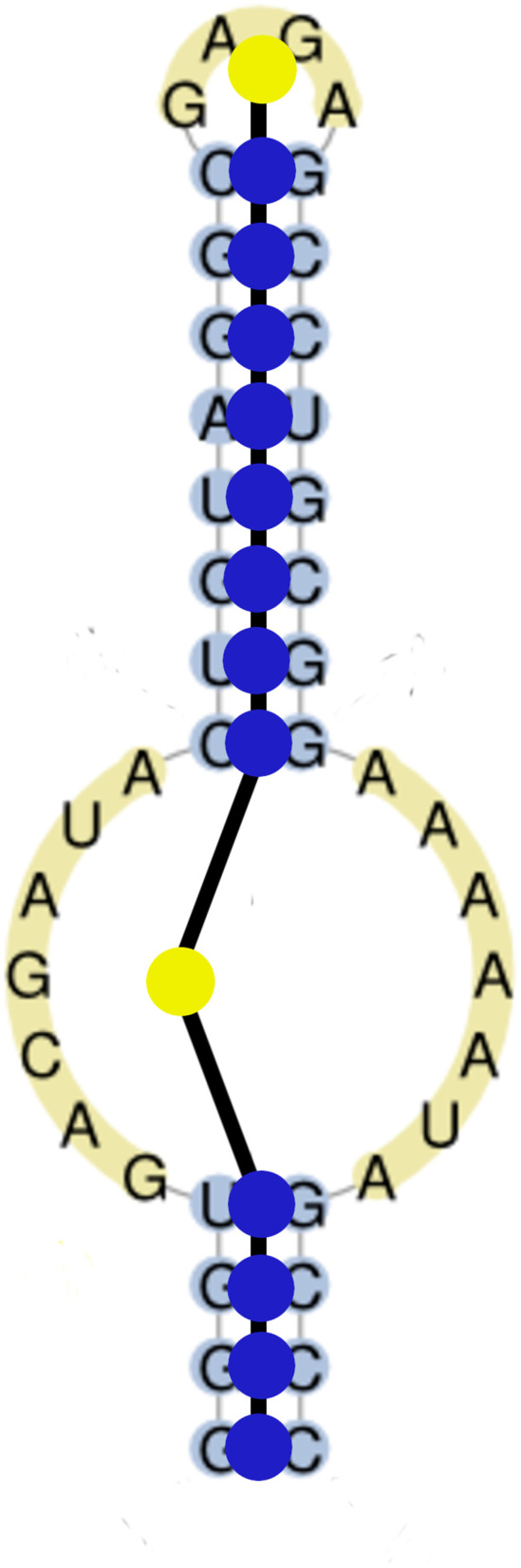
Representation of a GARN graph of a predicted molecule in 2D. Each base pair is represented by one vertex, while the 2-way junction is represented by one vertex, as it is in the study of GARN2 [10]. The PDB ID for this molecule is 1MNX.

**Fig 3:**
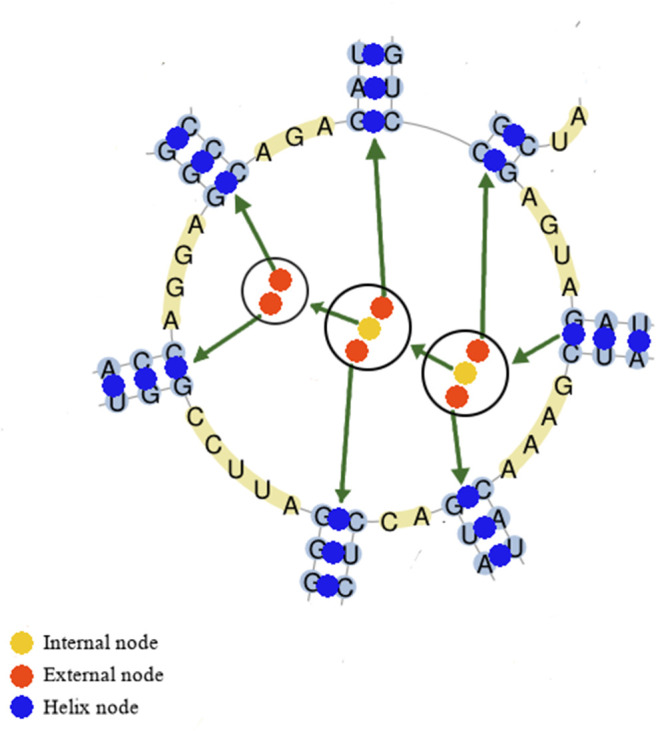
Graph representation of a seven-way junction. Each player has one predecessor and one or more successors, simplifying the folding process. External players (red) define the direction (arrows) of adjacent helices (blue), while internal nodes (yellow) position the next group of nodes (large circle). Each group contains two or three nodes.

- Each helix is represented by *j* vertices, where *j* denotes the number of base pairs in this helix, and each base pair is represented by a distinct vertex. Thus, a helix containing *j* base pairs is modeled as a chain of *j* consecutive vertices connected by edges. As mentioned earlier, this differs from the GARN/GARN2 model, in which several base pairs can be aggregated into a single vertex depending on helix length. These vertices will be referred to as base-pair vertices. An example is shown in Fig 1, where the molecule has more beads in the GARN3 model than in the GARN/GARN2 model.
- Terminal loops and 2-way junctions are represented by a single vertex (see Fig 2).
- 3-way junctions are represented by two vertices: one supporting the incoming base pair from the preceding vertex, and the other supporting the branching toward the two outgoing helices (one for each branch of the 3-way junction).
- Each higher-order *k*-way junction having *k* higher than 3 is represented by $k+\left\lfloor \frac{k-1}{2}\right\rfloor \;-2$ vertices (one player per vertex). We use two types of players to represent a *k*-way junction (see Fig 3):
  - k−1 **external players**, which connect the junction to adjacent helices;
  - $\left\lfloor \frac{k-1}{2}\right\rfloor \;-1$ **internal players**, which connect groups of external nodes.

To maintain a compact representation of the junction, two (and in some cases three) external players are collocated at the same spatial position.

The 3D molecule is represented as a graph embedded in space, where each player corresponds to a unique vertex (node); we use the terms player and node interchangeably in the remainder of the paper. An example of a complete GARN3 graph for a molecule with helices, terminal loops, and *k*-way junctions is provided in S1 Fig.

Another aspect of the 3D representation is the length of the edges, i.e., the distance between the vertices. Here, an edge connects two consecutive players along a given SSE, and additional edges connect SSE endpoints according to the secondary-structure connectivity (e.g., helix–junction or helix–loop adjacency). Each node is modeled as the center of a sphere representing the volume of the corresponding SSE. The length of an edge between two adjacent nodes is defined as the sum of their radii (i.e., half of their respective diameters) and is fixed.

For helix nodes representing a base pair, the edge length is set to 2.4 Å, following established parameters [10,47]. While previous versions shared this baseline, we model the helices at a finer granularity by representing each base pair as an individual player (vertex) on the graph. For other Secondary Structure Elements (SSEs), this study retains the edge length definitions from the original GARN model [10], where the distance between two adjacent vertices equals the sum of their radii. These diameters are either computed from statistical measurements within the reference set (yielding 5.6 Å or 11.2 Å depending on the SSE type) or derived from nucleotide counts using the 2.4 Å per base-pair spacing.

During development, we also explored an alternative representation with one bead per nucleotide, in which each nucleotide in the structure would be modeled as a separate player. This single-bead-per-nucleotide approach would have provided a simpler and more uniform way to represent all structural elements. However, after implementing and testing this model, we encountered significant limitations: the technique became computationally expensive, even for small molecules, and the resulting predictions showed poor structural accuracy compared to experimental structures. The primary issue is that each additional player increases the number of strategies to consider at each game turn, and, with regret-minimization algorithms, this quickly becomes computationally prohibitive. We therefore adopted the multi-bead helix representation described above, which provides a better balance between structural detail (compared to GARN2) and computational efficiency. More details about the single-bead model, including the machine learning scoring function we developed for it, are available in the S2 Appendix.

#### Conversion of all-atom PDB structures into the GARN3 representation.

For training and evaluation, native all-atom structures retrieved from the RCSB Protein Data Bank are converted into the GARN3 coarse-grained representation through a deterministic, hierarchical averaging rule. The basic operation, used at every level of the hierarchy, is the unweighted centroid:


$$\begin{array}{c} 𝐜\;=\;\frac{1}{m}\sum_{i=1}^{m}{𝐩}_{i}, \end{array}$$ (1)

where $\{{𝐩}_{1},\ldots ,{𝐩}_{m}\}\subset {\mathbb{R}}^{3}$ denotes the set of points to be aggregated. Only the type of the input points ${𝐩}_{i}$ changes from one level of the hierarchy to the next:

1. **From atoms to a single nucleotide.** For each nucleotide of the molecule, the input points are the heavy atoms of the nucleotide as read from the PDB file, and Equation (1) produces a single representative point for that nucleotide.
2. **From nucleotides to a player.** For helix base-pair, terminal-loop, 2-way junction, and 3-way junction players, the input points are the per-nucleotide representatives obtained at step 1 for the nucleotides aggregated by the player, and Equation (1) produces the player’s coordinates. The set of nucleotides assigned to a player follows the graph representation: the two nucleotides of the corresponding base pair for helix players, and the unpaired nucleotides of the corresponding SSE for terminal-loop and 2-way junction players.
3. **From sub-players to an internal *k*-way player (**k≥4**).** For internal players of *k*-way junctions, the centroid of Equation (1) is applied a second time, with *m* = 2, taking as input the two players already placed at step 2 on each side of the internal player:

$$\begin{array}{c} {𝐜}_{\text{internal}}\;=\;\frac{{𝐜}_{\text{mid}}+{𝐜}_{\text{edge}}}{2}, \end{array}$$ (2)

where **c**_mid_ and **c**_edge_ denote the coordinates of the two neighboring players on either side of the internal *k*-way junction player, as defined in the graph representation.

This conversion procedure is applied identically to all molecules used in the paper (parameter estimation, regression-model training, and test sets), so that all GARN3 coordinates compared in this work live in the same coarse-grained space. Nucleotides for which no heavy-atom coordinate can be read from the PDB file are simply omitted from step 1; molecules in which more than a small fraction of nucleotides cannot be assigned a representative point this way are excluded from both the analysis dataset and the test sets.

### 3.4. Game-theoretic modeling of RNA folding

GARN3 models RNA folding as a strategic game defined on the graph representation of the molecule. Each node (player) selects a spatial direction (strategy) to position its adjacent vertices. After each update, a scoring function evaluates the geometric compatibility between vertices. Strategy probabilities are then updated using regret-minimization algorithms.

#### Nash equilibrium and approximation principle.

The folding objective in this game-theoretic formulation is a Nash equilibrium: a configuration in which no player can improve its payoff by unilaterally changing strategy, that is, a stable folding of the molecule. Computing an exact equilibrium in this discrete and high-dimensional setting is generally infeasible (the implementation aspects of the approximation are detailed in Section 3.5); GARN3 therefore approximates one through iterative regret-minimization dynamics applied independently in each game.

#### Turn, game, and simulation.

We use the following terminology throughout the paper. A *turn* is the elementary step in which a single player (the active player of that turn) evaluates its possible strategies, selects one according to its regret-minimization algorithm, and uses it to update the position of the next player along the depth-first order; the positions of all further players in that order are then propagated accordingly. One turn therefore corresponds to exactly one strategy selection by exactly one player, and players take turns in the depth-first order. A *game* is a sequence of turns starting from a random initial configuration and ending either with a complete candidate structure or with an invalid configuration. A *simulation* of a given molecule consists of 500 independent games, each producing at most one candidate structure; the final prediction is selected from this pool of candidates using the sorting criterion of Section 5.

#### Strategies.

In the GARN3 model, a strategy corresponds to a direction in three-dimensional space along which a player positions the next connected node according to a predefined order. Angles are defined in a local reference frame oriented by the incoming edge (i.e., the direction of the previous edge), as in GARN2. Similarly to GARN and GARN2, the order of players in GARN3 is determined by a depth-first search starting from the largest junction (see S2 Fig).

After running multiple independent games, we select the best-scoring game outcome (i.e., the complete structure generated by a game, not a single turn) according to the sorting criterion.

Similarly to GARN and GARN2 [10,17], the set of possible strategies is available in an interval of 30° or 60°. However, to avoid an unnecessarily large number of strategies, the set of strategies for each SSE type is classified by junction type. That way:

- For 2-way junctions, two cases are distinguished, both using a discretization step of 30°:
- For bulges (internal loops in which one of the unpaired sides has length zero), the strategies range from 30° to 120°.
- In any other case, strategies range from 30° to 60°.
- For 3-way or higher-order junctions, the strategies vary from 0° to 180° by an interval of 60°.

To update the GARN3 strategies, we analyzed angular distributions derived from structures in our molecule database. To perform these analyses, the molecules in their native all-atom representations, obtained from the Protein Data Bank (PDB), were converted to the GARN3 model. After that, the angles between two players were calculated for different aggregations of SSE types. Angles in the histograms are expressed in radians.

**Fig 4:**
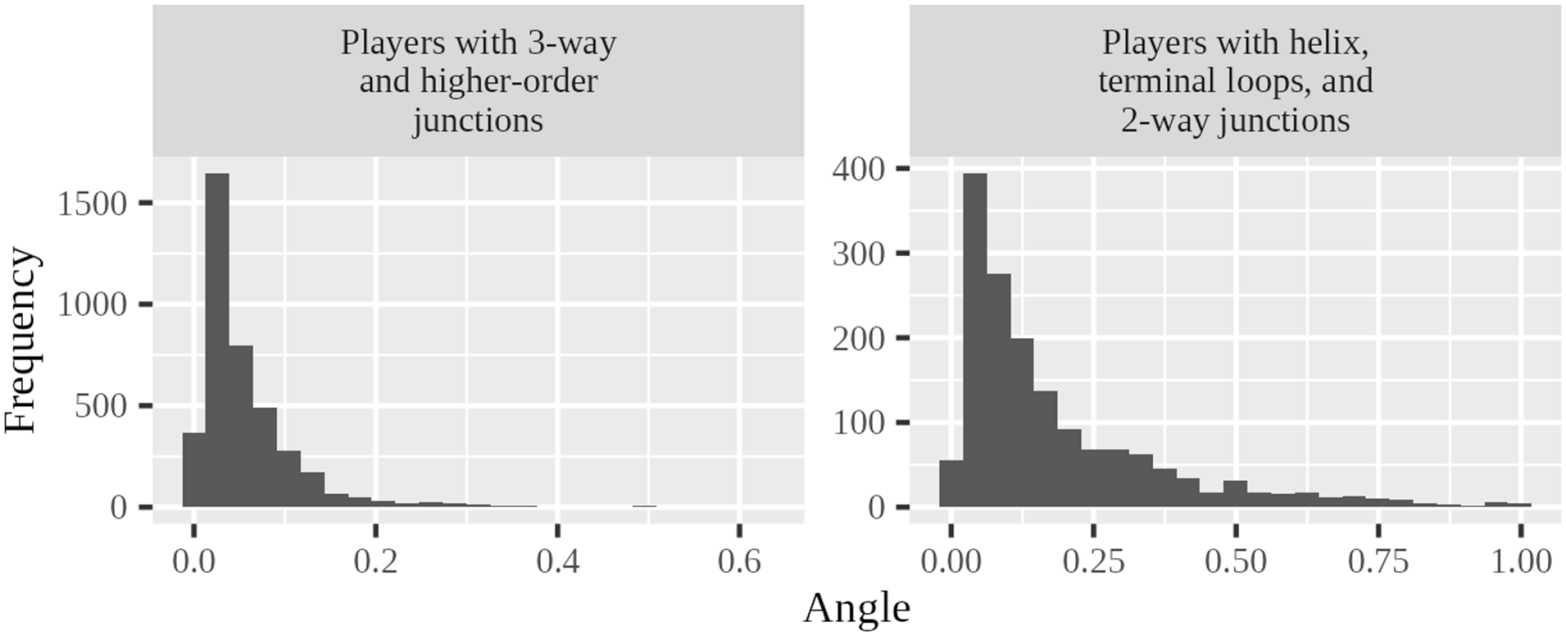
Angular distributions used to define the strategy sets for base-pair players. For each molecule in the dataset, base-pair players are identified, and the pairwise angles between consecutive players are computed, forming the basis for the strategy definition described in the text. Two base-pair players are considered part of the same helix, regardless of their position in the molecule, if they are connected to the same *k*-way junction. The left panel shows angle distributions for molecules with simpler topologies, containing only helices, terminal loops, and 2-way junctions. The right panel shows distributions for molecules with more complex topologies, including 3-way or higher-order junctions, highlighting how structural complexity influences the resulting strategy sets.

In Fig 4, the frequency distributions of angles are shown in two separate contexts: on the right, molecules containing 3-way or higher-order junctions; on the left, molecules restricted to helices, terminal loops, and 2-way junctions. Based on these distributions, the strategies for the base-pair player type were updated as follows:

- For molecules containing only Terminal (stem-loop), helices, and 2-way junctions: 0° – 30°;
- For molecules containing 3-way or higher-order junctions: 0° – 60°.

#### Initial configuration of a game.

Each game starts from a new initial configuration, built as follows. Players are placed sequentially in the depth-first order described in the Strategies paragraph above, starting from the highest-order junction (i.e., the *k*-way junction with the largest *k* present in the molecule; ties broken by sequence index). The first player in this order is placed at the origin of ${\mathbb{R}}^{3}$ with a fixed canonical orientation of the local reference frame in which subsequent strategies are expressed.

Each subsequent player *i* independently samples a strategy $\sigma_{i}$ uniformly at random from its set of admissible strategies, the admissibility set being determined solely by the SSE type of *i* (see the Strategies paragraph above). Recalling that $\sigma_{i}$ specifies the direction along which player *i* positions its successor in the local frame, the position of the successor is then computed deterministically from the position of *i*, the direction of the incoming edge of *i*, the sampled angle $\sigma_{i}$, and the fixed edge length associated with the SSE types of the two endpoints (Section 3.3).

If the resulting layout contains a pair of intersecting edges, the initial draw is rejected and resampled with new independent strategies, so that every game starts from a geometrically valid configuration. This is the same validity criterion used to detect early termination during the game itself (see Section 3.5).

The 500 independent games of a simulation use 500 independent draws of these initial strategies. The corresponding pseudo-random generator and its seed are documented in the public source distribution, so that any reported simulation can be reproduced.

### 3.5. Computing a nash equilibrium

Computing an exact Nash equilibrium in the discrete and high-dimensional game induced by the GARN3 representation is computationally intractable [48]. For this reason, GARN3 relies on regret-minimization algorithms to approximate stable configurations.

Regret minimization provides a principled framework for repeated decision-making under uncertainty. In repeated games, no-regret dynamics are known to converge to correlated equilibria and, in certain settings, to approximate Nash equilibria. In our context, this approach enables each player (a pseudoatom) to iteratively refine its spatial strategy based on geometric compatibility with neighboring players.

Using the same approach as in GARN/GARN2 [10,17], the regret-minimization algorithms EXP3 *(Exponential-weight algorithm for Exploration and Exploitation)* [49] and UCB *(Upper Confidence Bound)* [50] were implemented in GARN3. According to the analysis in the study by GARN [17], EXP3 yielded better predictions for molecules with 3-way or higher-order junctions, whereas UCB performed better in the other cases. In this paper, we analyze whether the prediction performance, classified by molecule type and using a specific regression minimization algorithm, follows the same pattern observed in the GARN [10] study; in other words, whether EXP3 remains more effective for 3-way or higher-order junctions and UCB for the remaining cases.

Using the same approach as in GARN/GARN2 [10,17], the implementation of the regret-minimization algorithms in GARN3 proceeds as follows:

1. At the start of the game, all players select an initial strategy to construct a preliminary 3D structure.
2. They act sequentially, taking turns according to the regret-minimization algorithm. On its turn, a player evaluates the scores (representing the sum of all molecular interactions) for each of its possible choices, selects one, and immediately updates its positioning for subsequent players.
3. If the configuration becomes invalid at any stage, the process terminates, and the participants are penalized. In GARN3, a configuration is considered invalid when two edges intersect.

A game terminates under one of two conditions:

- **Successful termination:** every player has updated its strategy 30 times. The structure obtained at the end of this sequence constitutes the candidate output of the game.
- **Early termination:** the configuration becomes invalid at some point during the game. In GARN3, a configuration is considered invalid when two edges of the graph intersect in ${\mathbb{R}}^{3}$. In this case, the game is aborted, all players are penalized, and no candidate structure is produced.

We do not impose any additional convergence criterion (such as a stability threshold on the score), since the stochastic regret-minimization dynamics are not guaranteed to converge pointwise to a fixed strategy profile. Robustness across random initializations is achieved by running 500 independent games per molecule and aggregating their outcomes.

The process is repeated for multiple game instances, following a multi-armed bandit paradigm. The final predicted structure corresponds to the best-performing game according to the selected sorting criterion.

### 3.6. Scoring

To define the probability of each possible strategy, a scoring function is applied after each game turn, using a KB (knowledge-based) approach to the distance between atoms in a molecule. In this KB function, the interaction between two players *i* and *j* is modeled using a Lennard–Jones-type potential [19], defined as


$$\begin{array}{c} -A_{i,j}\times \left({\left(\frac{B_{i,j}}{d}\right)}^{12}-2\times {\left(\frac{B_{i,j}}{d}\right)}^{6}\right), \end{array}$$ (3)

where *d* denotes the Euclidean distance between players *i* and *j*. The parameters $A_{i,j}$ and $B_{i,j}$ depend on the respective types of the interacting players (helix or junction).

Two approaches were used to define the parameters $B_{i,j}$:

1. Static values derived from statistical analysis of the training dataset,
2. values predicted using a machine learning regression model.

#### 3.6.1. Scoring static parameters.

To update the scoring parameters, we analyzed distances between atoms in molecules from the database used in this study. To perform these analyses, the molecules in a native all-atom representation obtained from PDB files were transformed into the GARN3 model, as in the analysis to update the strategies. Afterward, the distance calculations were performed using different aggregations of the SSE types. For each updated score, the value corresponds to the best distance in the histogram; in other words, the one with the highest frequency. The metric used to represent the distances between atoms or structures in this paper is angstrom (Å), where 1 Å = 10^−10^*m*.

**Fig 5:**
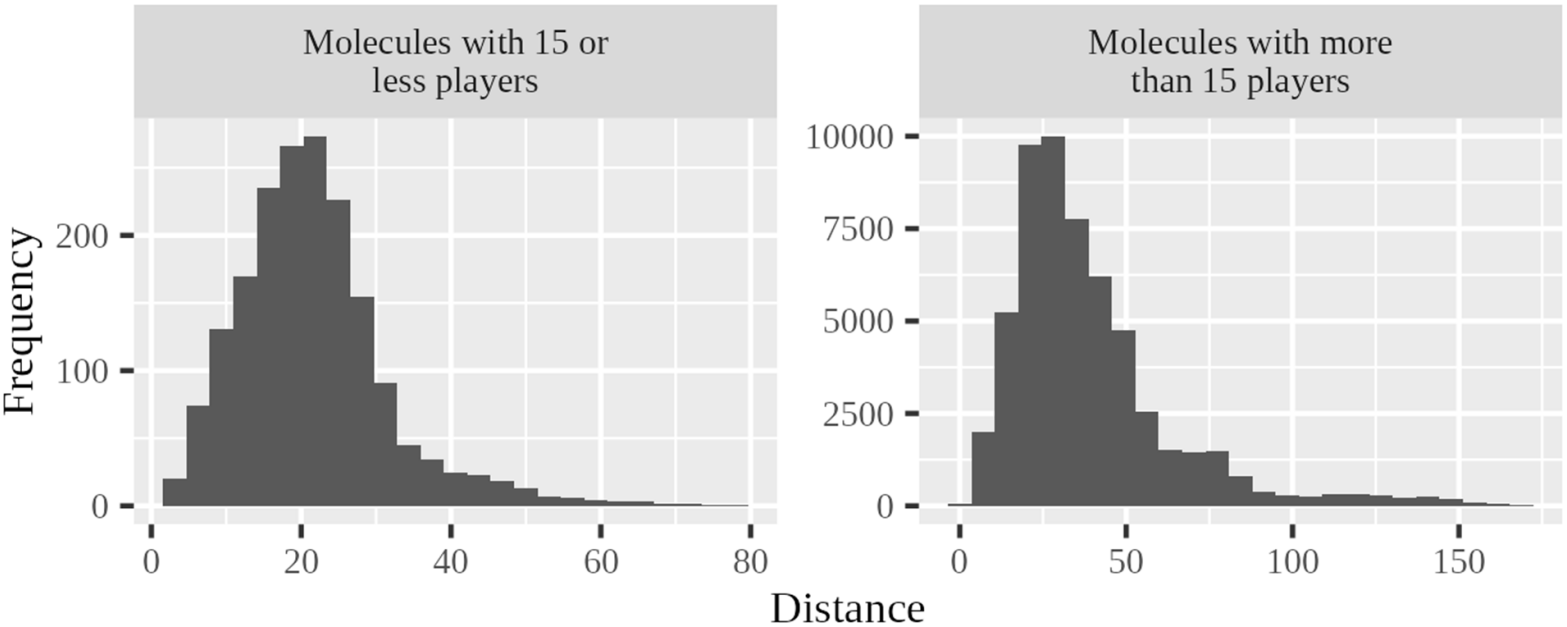
Scores for interactions between two base-pair players, regardless of their placement within the structure. Therefore, the molecules in our dataset are included, and all the base-pairs within the structures are considered.

For an interaction between two base-pair players, the results are shown in Fig 5, with molecules of at most 15 players on the left and molecules of more than 15 players on the right. The threshold value of 15 was selected empirically after testing every integer cutoff between 1 and 30: for each candidate cutoff, the resulting two distributions were fitted to a Lennard–Jones potential (the functional form used in the scoring function of Equation (3)), and the cutoff at 15 players yielded the closest agreement between the empirical distributions and the fitted Lennard–Jones curve on both sides of the split. Splitting the dataset at this threshold therefore isolates two regimes in which the scoring assumption used by GARN3 is best supported, while a single distribution pooling all molecules together would mix short and long structures into a shape that deviates more substantially from the Lennard–Jones form.

**Fig 6:**
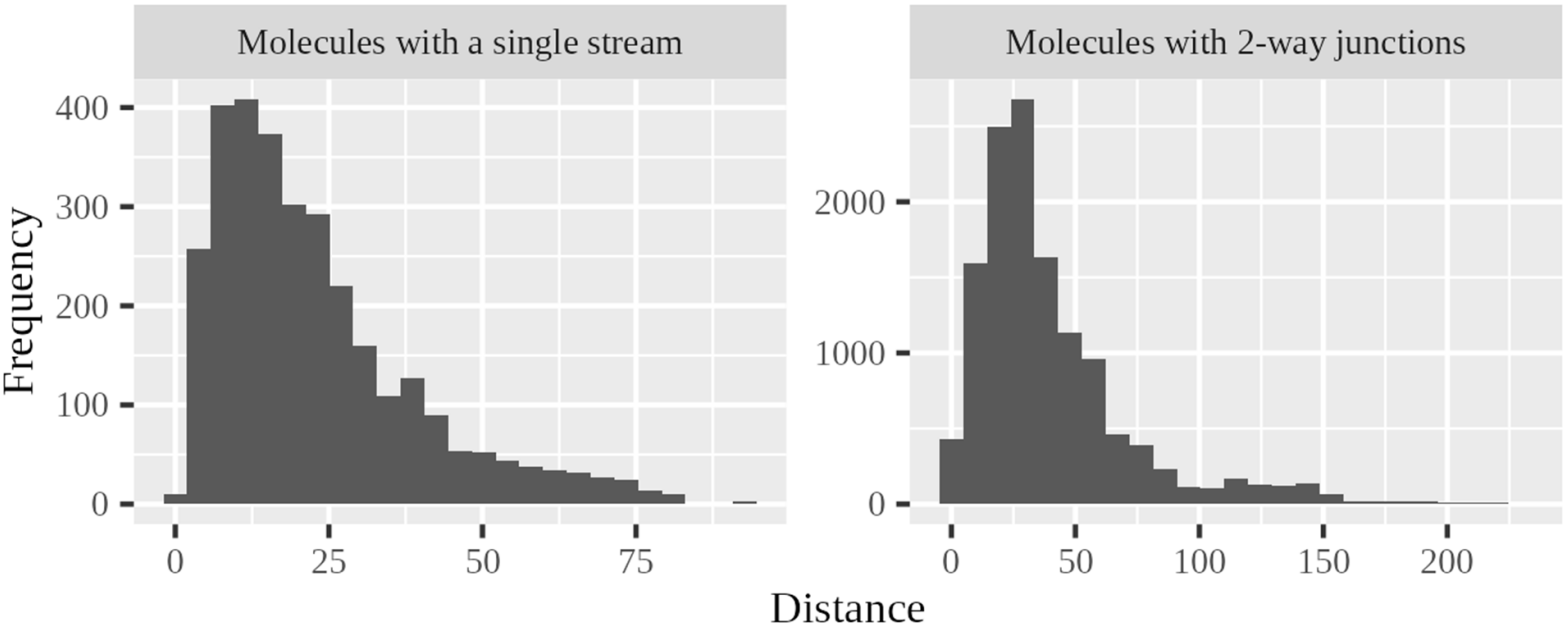
Scores for interactions between a base-pair player in a helix and an unpaired region player. The normal distribution on the left side is filtered with molecules in our dataset that contain an unpaired region at the start or at the end of the structure (i.e., opening or closing). On the right side, the only filter criterion is the *k*-way junction topology, where molecules with 3-way or higher-order junctions are excluded.

For interactions between players of the helix and 2-way junction SSE types, the results are shown in Fig 6. On the left side of the figure, only molecules containing a single strand are considered; on the right side, molecules containing 2-way junctions are considered.

After completing the molecular data analysis, some scoring function parameters were updated. These are related to the base-pair player that interacts with other SSE types. The updated static parameters are as follows:

- Two base pair players interacting: 18 Å, if the molecule has at most 15 players; 23 Å otherwise.
- A base pair and an unpaired stream player: 14 Å if the unpaired is a single strand; 26 Å if it is a terminal loop.
- A base pair and a 2-way junction player: if the molecule has only stem-loop, base pair, and 2-way SSE types, 11 Å; or 22 Å otherwise.

For the other interactions between SSE types, this paper follows the previous settings of the GARN2 study [10].

#### 3.6.2. Regression model for interaction distance estimation.

A regression model was trained to predict optimal interaction distances. To obtain more accurate distance estimates for use in the GARN3 scoring function, different machine learning algorithms were evaluated for regression. These include: Gradient Boosting, Decision Trees, Multilayer Neural Networks, LASSO, Ridge, Random Forests, Linear Regression, k-Nearest Neighbors, and SVM. The features used in these algorithms are described below. They represent characteristics of the SSE of a molecule; some features consider the entire molecule, while others focus only on the interaction between two elements. These elements are players in the GARN model.

To train and test these models, the molecules in the set were first converted to the GARN 3D structure representation, which uses pseudoatoms rather than the original all-atom coordinates. Therefore, the resulting database consists of a set of GARN structures, where the coordinates of these players correspond to those of the molecules obtained from the RCSB Protein Data Bank. The cross-validation method *k-fold* was applied using 10 folds, a popular choice that is likely to be accurate [51].

To define the features, the literature was reviewed to understand the potential reusability of aspects of similar studies. Some recent studies reviewing the state of the art in RNA prediction using machine learning [52–55] describe the advances; however, the results of these models are not comparable to those needed in this study. In addition, the input to these models considers various aspects of the study’s context.

An investigation of characteristics relevant to determining the distance between two GARN players was conducted, considering SSE types and players’ placement within the GARN3 graph representation. Interactions are always defined between two players. The utility function of a player corresponds to the sum of the scoring-function values computed over its interactions with all other players. The scoring function, therefore, depends on the types of interacting players, their spatial distance, and other structural features. The parameters of the scoring function between two players, denoted *X* and *Y*, are learned through a machine learning regression model. The relevant features defined are as follows:

1. SSE type of player *X*
2. SSE type of player *Y*
3. Shortest-path distance between players *X* and *Y*, considering that the GARN3 model is represented as a graph. For that, the Dijkstra algorithm [56] was used.
4. Quantity of base pairs inside the *k*-way junction, in case players *X* and *Y* are two base pairs at the same branch in a *k*-way junction. Otherwise, this value is zero;
5. If players *X* and *Y* are placed at the same *k*-way junction, this feature has a value of 1; otherwise, its value is zero;
6. If players *X* and *Y* are pseudoknots interacting (kissing loops), this value is 1; otherwise, its value is zero;
7. If players *X* and *Y* are a base pair and a bulge, this value is 1, and 0 otherwise;
8. Largest junction quantity in the molecule. For example, if the molecule has only base pairs and a stem-loop, this number is 1. If the molecule has a 2-way junction, this number is 2, and it follows the same logic for higher-order *k*-way junctions;
9. If this is an interaction between two players *X* and *Y* that are *k*-way junctions, having *k* > 1, captures the length of the smallest side of this junction; otherwise, this value is −1;
10. If this is an interaction between two players that are *k*-way junctions, having *k* > 1, captures the length of the largest side of this junction; otherwise, this value is −1;
11. Total of players considering the entire molecule.

The regression model outputs the optimal distance between two players based on the input features.

### 3.7. Sorting criteria: Selection of the final structure

GARN3 generates multiple candidate structures; at this stage, no final structure has yet been selected. The GARN3 algorithm should choose one. In the GARN2 study [10], three approaches were presented: (i) sorting by global score, where the sum of all players’ scores is taken; (ii) sorting by minimum score, where the molecule with the lowest score is selected under the assumption that it has incurred less penalization; and (iii) sorting by maximum distance, where the maximum distance between players is used as the selection criterion.

From these three criteria, the maximum-distance criterion is adopted in this work, as it was identified as the most relevant in the GARN2 study. Accordingly, for each candidate structure, the maximum distance-based penalty across all relevant interacting pairs is computed, and the structure minimizing this maximum is selected. This ranking criterion is further validated through simulations on the test set.

### 3.8. Evaluation of molecules

For each molecule, a widely used metric in the literature, namely RMSD (Root Mean Squared Deviation) [45] was used. This metric measures the average distance between two molecular structures, accounting for each element within them. The RMSD is defined by


$$\begin{array}{c} RMSD(a,b)=\sqrt{\frac{1}{p}\sum_{i=1}^{p}{\left\|a_{i}-b_{i}\right\|}^{2}} \end{array}$$ (4)

where *a* and *b* are two molecules, *p* is the number of elements (which is the number of players in GARN), *i* is the molecule, and $a_{i}$ and $b_{i}$ are the position of the player *i* in the molecules *a* and *b*.

Additionally, the evaluation considers another metric commonly used in the literature, namely the TM-score (Template Modeling score). The TM-score assigns greater weight to smaller distance errors than to larger ones [46]. The resulting value ranges between 0 and 1, where 1 indicates a perfect structural match between the compared structures. The TM-score is defined as follows:


$$\begin{array}{c} \text{TM-score}(a,b)=\max\left[\frac{1}{L}\sum_{i=1}^{L}\frac{1}{1+{\left(\frac{\left\|a_{i}-b_{i}\right\|}{d_{0}(L)}\right)}^{2}}\right] \end{array}$$ (5)

where *L* is the number of elements (which is the total of players in the GARN structure), ‖·‖ denotes the Euclidean norm, and *d*_0_(*L*) is the length-dependent normalization factor defined by $d_{0}(L)=1.24(L-15{)}^{1/3}-1.8.$

In Section 2, several techniques reported in the literature were mentioned that aim to predict 3D structures of RNA molecules. To support our discussion of the GARN3 simulation results, we selected 12 representative methods from the literature: four template-free techniques (iFoldRNA [5], NAST [6], SimRNA [9] and IsRNA1 [11]); six template-based techniques (FARFAR2 [12], RNAComposer [13], VFoldLA [25], 3dRNA [16], FebRNA [27] and MC-Sym [24]); and two deep learning techniques (AlphaFold 3 [3] and trRosettaRNA [36]). Although GARN and GARN2 generate molecules with fewer players than GARN3, GARN2 is used for comparison because it is closely related to this study.

## 4. Learning-based interaction distance estimation results

Several evaluation metrics were used to assess the machine learning regression models. To guide feature selection and interpret model behavior, the SHAP (SHapley Additive exPlanations) method [57] was applied. This method numerically quantifies the importance of each input feature in the prediction model. After refining features using SHAP, the most relevant features are described in the following section.

After defining the model features, further analysis was conducted to identify an appropriate machine learning algorithm for estimating interaction distances used in the scoring function. Although several evaluation metrics are available in the literature, MAE and RMSE are among the most commonly used to assess model performance [58,59]. The MAE (Mean Absolute Error) measures the average absolute difference between predicted and observed values, regardless of outliers or high values. For example, an MAE of 10 in our model indicates an average deviation of 10 Å between predicted and observed distances. Therefore, if the MAE is 10 in our model, it means we have an average difference of 10 Å between the predicted and actual values. The RMSE (Root Mean Squared Error) metric is the square root of the MSE (Mean Squared Error), which calculates the squared error and gives more weight to large errors. The difference between RMSE and MSE is the scale: MSE is expressed in squared units, whereas RMSE returns the error to the original scale of the predicted quantity.

**Fig 7:**
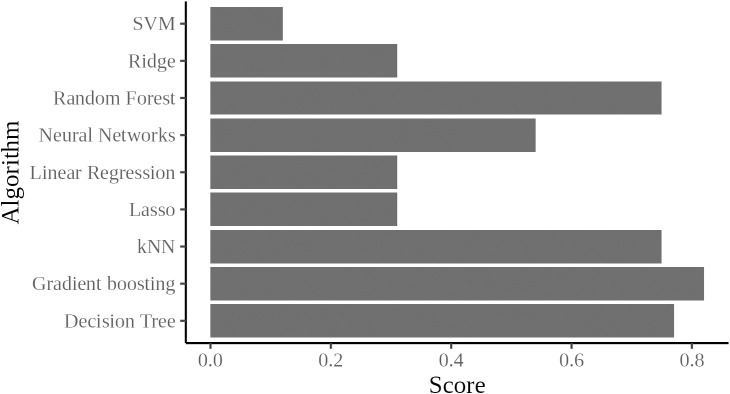
Comparison of regression models on the interaction-distance dataset constructed from the converted RNA structures, where each sample corresponds to a pair of players with extracted features and a target optimal distance. The score from the results of k-fold evaluation described is R^2^.

In our experiments, Gradient Boosting demonstrated better performance over the other algorithms, as illustrated in Fig 7. After running the *k*-fold cross-validation, the average performance metrics were approximately 11 Å, and the *R*^2^ (R Squared) metric was also evaluated. The *R*^2^ metric takes a value between 0 and 1 and measures the proportion of variance in the observed data explained by the model. The closer to 1, the better the model. For the model in this study, *R*^2^ has the value ≈0.82. The model validation metrics are presented in S3 Table.

**Fig 8:**
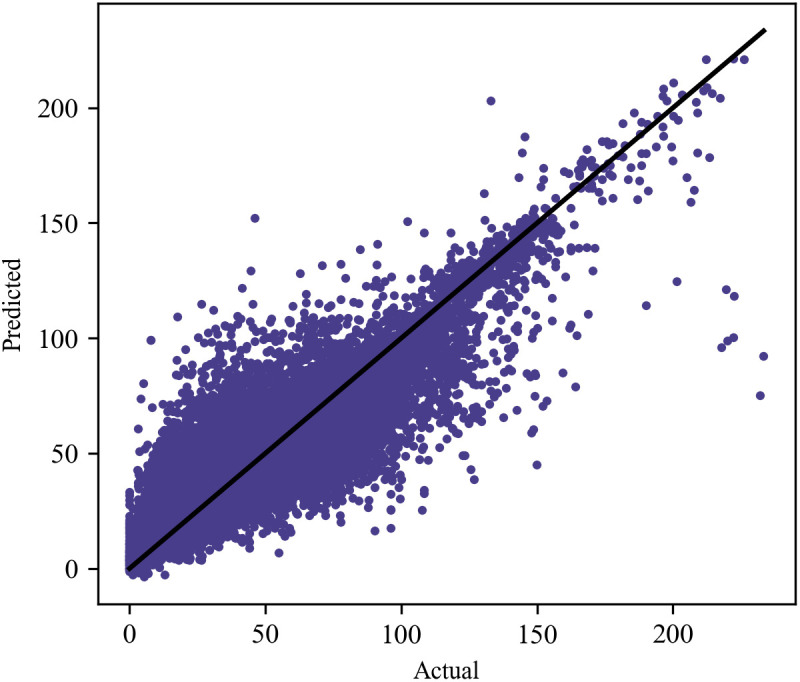
Comparison of real and predicted values using the Gradient Boosting regression model, where the output is a distance between 2 players. For each point in the plot, the x-axis is defined by the expected value from the dataset, and the y-axis is defined by the predicted value from the model.

An analysis of the prediction error was also performed using the Gradient Boosting algorithms trained on our database, as illustrated in Fig 8. In this figure, the predicted distances are compared with the actual (expected) distances. The diagonal line represents perfect agreement, and a strong correlation is observed when the predicted values closely match the actual values.

Since the integration of machine learning in the scoring function differs from previous GARN studies [10,17], it is important to understand whether the new approach performs as intended. Hence, simulations were performed using Test Set A (see S1 Table), applying the scoring function with both static parameters and parameters predicted by the regression model.

**Fig 9:**
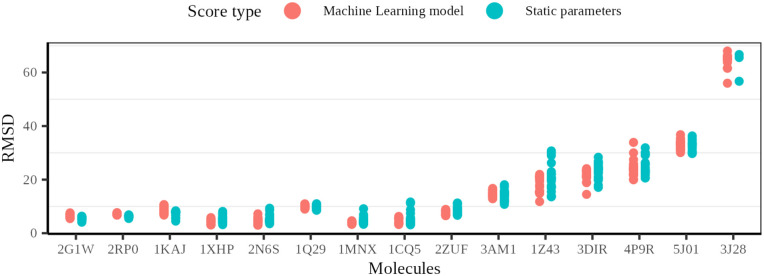
Comparison between simulations using scoring function with static parameters and scoring function with machine learning model. The molecules not present in this graphic were not able to be obtained using the static parameters.

The aggregated results for Test Set A are shown in Fig 9. In some cases, simulations using static parameters did not produce valid structures; these cases are not displayed in the figure. Although machine learning-based parameters did not consistently outperform static parameters, they led to lower RMSD values for the majority of molecules in the test set. In addition, in a subset of cases, valid predictions were obtained only with the learned parameters, whereas simulations with static parameters did not produce valid structures, as seen in Fig 9. For molecules that were not successfully predicted using static parameters, the issue was that the optimization algorithm became exhausted and generated invalid configurations (e.g., edge intersections), resulting in early termination without producing a structure. Therefore, the simulations in the next section use parameters from the machine learning model.

## 5. Simulation results

This section presents the results of the experiments, showing the best results from the molecule simulations (i.e., the lowest RMSD) using GARN3 and applying the two regret-minimization algorithms, EXP3 and UCB. Afterward, we compare GARN3 with the other selected techniques, report execution times, and analyze the results obtained after ranking and selecting the best structure at the end of the games performed for each molecule. For each comparison technique, enough simulations were performed to obtain 20 structures per molecule. In practice, when a method allowed the generation of *n* structures per execution, we performed ⌈20/n⌉ independent prediction runs for the same molecule.

The simulations were run using the test sets (see S1 Table and S2 Table) in this study, applying different scoring functions. The results are described and compared with other techniques in the literature. Considering the number of techniques compared in this study, the results of the comparisons with other techniques are organized into three categories for better visualization of tables and figures: template-based, template-free, and deep learning. In addition, in most tables and figures in this section, the molecules in the test set are also divided into categories, based on the SSE types, as follows:

- **2-way junction molecules**, whose junctions have a maximum of 2 ways;
- **3-way junction molecules**, whose junctions have a maximum of 3 ways;
- ***k*-way junction molecules**, which have at least 4-way or higher-order junctions;
- **Pseudoknot molecules**, which have at least 1 pseudoknot.

When requesting simulations for the 3dRNA web server, 3dRNA-Lib2 was used for the predictions, with the other parameters set to their default values. For all the techniques tested, the same secondary structures used in the GARN simulations were provided as input, except for those that do not allow secondary structure specification. In this scope, the ones that do not allow are trRosettaRNA and AlphaFold 3. NAST simulations were performed using 5 000 steps, and IsRNA1 was run with 20 000 000 steps (default setting). VFoldLA was executed using its default parameter settings.

For techniques that do not provide a web server, the simulations were run on a local machine with an Intel Core i7-12800H 12th-generation processor at 2.4 GHz and 64 GB of physical RAM, with Windows 11 (version 24H2) installed.

### 5.1. Regret minimization algorithms

As mentioned before, the regret-minimization algorithms applied in GARN3 are UCB and EXP3. The motivation for using UCB was to understand how the behavior is because, in the GARN study [10], smaller molecules are better predicted using UCB. In the GARN3 model, because there are more players (pseudoatoms) in the final 3D structure for the same molecules, UCB could behave slightly differently.

Among the tested molecules, smaller ones, usually with simpler topologies and at most 2-way junctions, yielded better results when simulations were run with UCB. Within this subset, 3 of the 5 molecules achieved lower RMSD values with UCB. In addition, among the molecules with pseudoknots, even though their differences were smaller (less than 1 Å), the results were also better with UCB.

For larger molecules with 3-way or higher-order junctions, better results were obtained when using EXP3. Another point to consider was the time required to predict, for example, the molecule 1Q29, which has 3-way junctions: it took approximately 20 hours with UCB, whereas EXP3 returned better results in less than 1 minute. All results are reported in S8 Table and S9 Table.

### 5.2. Simulations from other techniques

**Fig 10:**
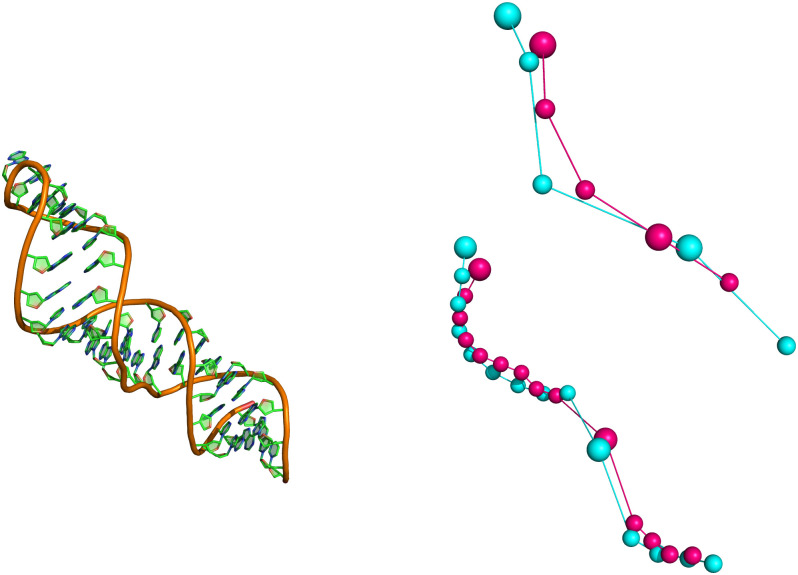
Comparison of GARN2 and GARN3 structural representations using PDB ID 1MNX. Left: Complete experimentally determined structure. Right: Magnified views of the GARN2 (top) and GARN3 (bottom) representations, illustrating the increased pseudoatom density in the GARN3 model. Native experimental structures, converted to the GARN2 and GARN3 coarse-grained representations, are shown in cyan, whereas simulation-derived models are depicted in pink.

Unlike the others, the comparison with GARN2 was conducted using a different approach because the final 3D structure contained a considerably smaller number of pseudoatoms. Starting from the original PDB molecule, a GARN2 model is generated, with fewer pseudoatoms than GARN3. Using the same molecule, a GARN3 model is generated. The GARN2 model is used to compare GARN2 simulation results, whereas the GARN3 model is used for GARN3 results and for other techniques in the literature. For the other techniques, the predicted structure (usually a PDB file) is converted into the GARN3 representation and used for comparison. Fig 10 illustrates both models: the original structure is shown on the left, the GARN2 model in the upper right, and the GARN3 model in the lower right. For all other techniques, the GARN3 representation was used for comparison. There is an example of comparison in S3 Fig with the molecule 1XHP, where the best structure predicted by each of the techniques is represented, compared to its native structure.

After running the simulations with GARN3 and other techniques, the minimum and maximum RMSD values for each molecule and method are reported in Table 1 (results on Test Set A) and Table 2 (results on Test Set B), considering only template-free modeling techniques. In these tables, for each molecule, the best result obtained with either EXP3 or UCB is reported, and the scoring parameters correspond to those predicted by the machine learning model. For each molecule and each method, we report the minimum and maximum RMSD obtained across the 20 generated structures (rows “Min” and “Max”). The TM-score reported in each row corresponds to the best-ranked and worst-ranked structures by TM-score across the runs.

**Table 1:** Comparison with template-free modeling techniques, using Test Set A. The model for comparison in GARN2 was different from the others due to the number of pseudoatoms in its predictions, which is lower than GARN3. The highlighted values in each row aim to help the visualization of the top-ranked technique, considering the lowest RMSD and the highest TM-Score. The RMSD and TM-Score in the cells were obtained independently; therefore, the structure with best-ranked RMSD not necessarily is the one with best-ranked TM-Score.

Mol.	Type	Len.	RMSD /TM	iFoldRNA	NAST	SimRNA	IsRNA1	GARN2	GARN3
1XHP	2-way	32	Min	1.13 / 0.909	8.51 / 0.538	–	**1.12** / 0.908	2.15 / 0.834	3.1 / 0.698
			Max	**1.13** / 0.909	8.51 / 0.538	–	1.43 / 0.893	4.85 / 0.604	5.83 / 0.578
1MNX	2-way	42	Min	2.99 / 0.662	9.84 / 0.458	2.42 / 0.719	**1.47** / 0.898	6.96 / 0.568	3.29 / 0.592
			Max	15.4 / 0.425	9.84 / 0.458	5.35 / 0.404	**3.39** / 0.618	9.56 / 0.343	4.63 / 0.499
1CQ5	2-way	43	Min	2.93 / 0.668	9.18 / 0.300	–	3.4 / 0.681	**2.62** / 0.741	3.25 / 0.617
			Max	10.2 / 0.470	9.18 / 0.300	–	**5.58** / 0.608	8.68 / 0.502	6.26 / 0.530
2RP0	2-way	27	Min	2.59 / 0.865	4.59 / 0.835	**0.897** / 0.956	4.21 / 0.915	5.51 / 0.801	6.76 / 0.774
			Max	2.59 / 0.865	4.59 / 0.835	**1.58** / 0.911	7.47 / 0.868	11 / 0.798	7.68 / 0.752
2N6S	2-way	36	Min	–	6.85 / 0.579	2.10 / 0.799	**2.05** / 0.792	–	3.01 / 0.627
			Max	–	6.85 / 0.579	3.64 / 0.603	**2.05** / 0.792	–	7.22 / 0.601
1Q29	3-way	41	Min	7.28 / 0.414	–	**2.00** / 0.789	2.05	4.20 / 0.420	9 / 0.354
			Max	8.68 / 0.380	–	6.87 / 0.412	**2.05**	10.2 / 0.319	11 / 0.421
3DIR	3-way	174	Min	**13.7** / 0.373	24.1 / 0.283	–	–	16.2 / 0.334	14.5 / 0.304
			Max	**21.0** / 0.324	24.1 / 0.283	–	–	29.9 / 0.271	24.1 / 0.268
4P8Z	3-way	188	Min	18.5 / 0.313	**15.9** / 0.361	–	23.3	–	18.6 / 0.380
			Max	25.2 / 0.239	**15.9** / 0.361	–	23.3	–	27.5 / 0.234
3AM1	3-way	81	Min	11.4 / 0.475	8.29 / 0.371	**8.23** / 0.413	–	18.4 / 0.361	12.9 / 0.350
			Max	16.5 / 0.393	**8.29** / 0.371	14.4 / 0.359	–	19.3 / 0.254	16.7 / 0.342
4RZD	3-way	102	Min	12.5 / 0.344	–	–	**1.22** / 0.385	8.12 / 0.361	10.1 / 0.376
			Max	16.0 / 0.312	–	–	**14.1** / 0.385	15.8 / 0.391	16.4 / 0.377
4QKA	3-way	122	Min	10.4 / 0.424	73.2 / 0.235	–	**9.93** / 0.431	12.6 / 0.335	11.2 / 0.382
			Max	12.4 / 0.313	73.2 / 0.235	–	**9.93** / 0.431	23.0 / 0.314	19.9 / 0.357
1Z43	3-way	101	Min	**4.22** / 0.551	20.3 / 0.371	–	14.8 / 0.354	11.0 / 0.420	11.8 / 0.348
			Max	**12.7** / 0.304	20.3 / 0.371	–	14.8 / 0.354	29.3 / 0.310	22 / 0.319
4P9R	3-way	189	Min	27.3 / 0.377	29.9 / 0.313	–	**14.5**	18.5 / 0.332	19.9 / 0.297
			Max	30.1 / 0.293	29.9 / 0.313	–	**14.5**	27.7 / 0.211	33.9 / 0.237
4OQU	*k*-way	97	Min	10.7 / 0.353	26.2 / 0.296	**5.32** / 0.445	–	10.5 / 0.400	11.6 / 0.348
			Max	14.3 / 0.324	26.2 / 0.296	**5.32** / 0.445	–	20.7 / 0.295	18.2 / 0.277
4QK8	*k*-way	124	Min	11.2 / 0.423	18.7 / 0.344	13.2 / 0.396	–	15.5 / 0.353	**9.43** / 0.344
			Max	**14.5** / 0.356	18.7 / 0.344	16.5 / 0.313	–	23.4 / 0.259	19.9 / 0.355
5J01	*k*-way	418	Min	54.1 / 0.110	230.0 / 0.025	–	–	35.1 / 0.229	**30.1** / 0.165
			Max	59.2 / 0.055	230.0 / 0.025	–	–	53.1 / 0.131	**36.8** / 0.157
3J28	*k*-way	1533	Min	97.8 / 0.059	77.0 / 0.039	–	–	58.9 / 0.080	**53.2** / 0.054
			Max	99.8 / 0.051	77.0 / 0.039	–	–	78.6 / 0.039	**68** / 0.056
1C2W	*k*-way	2904	Min	–	–	–	–	69.1 / 0.044	**67.1** / 0.038
			Max	–	–	–	–	85.4 / 0.030	**82.4** / 0.025
2NBX	*k*-way	108	Min	–	22.7 / 0.393	**9.75** / 0.432	17.2 / 0.367	58.1 / 0.162	16 / 0.303
			Max	–	22.7 / 0.393	**16.7** / 0.371	17.2 / 0.367	74.4 / 0.130	20.8 / 0.369
2G1W	pseudo-knot	22	Min	2.64 / 0.701	2.76 / 0.779	**2.05** / 0.845	2.14 / 0.785	5.81 / 0.802	5.46 / 0.834
			Max	2.64 / 0.701	2.76 / 0.779	2.90 / 0.738	**2.14** / 0.785	11.4 / 0.801	7.59 / 0.810
1KAJ	pseudo-knot	32	Min	**3.26** / 0.795	4.25 / 0.844	4.12 / 0.868	4.26 / 0.878	6.74 / 0.641	6.77 / 0.776
			Max	**3.26** / 0.795	4.25 / 0.844	4.21 / 0.852	4.26 / 0.878	10.6 / 0.639	10.7 / 0.779
2ZUF	pseudo-knot	78	Min	11.7 / 0.403	10.2 / 0.378	–	15.4 / 0.381	7.07 / 0.521	**6.6** / 0.359
			Max	16.2 / 0.357	10.2 / 0.378	–	15.4 / 0.381	12.3 / 0.349	**8.88** / 0.391

**Table 2:** Comparison with template-free modeling techniques, using Test Set B. The model for comparison in GARN2 was different from the others due to the number of pseudoatoms in its predictions, which is lower than GARN3. The highlighted values in each row aim to help the visualization of the top-ranked technique, considering the lowest RMSD and the highest TM-Score. The RMSD and TM-Score in the cells were obtained independently; therefore, the structure with best-ranked RMSD not necessarily is the one with best-ranked TM-Score.

Mol.	Type	Len.	RMSD /TM	iFoldRNA	NAST	SimRNA	GARN2	GARN3
8VQV	2-way	64	Min	**6.43** / 0.53	8.56 / 0.408	7.51 / **0.538**	–	8.05 / 0.418
			Max	10.51 / **0.452**	**8.56** / 0.408	13.53 / 0.347	–	11.3 / 0.362
8VVJ	2-way	64	Min	**3.97** / 0.652	16.3 / 0.439	4.63 / **0.756**	–	9.02 / 0.493
			Max	19.87 / 0.367	16.3 / 0.439	13.48 / 0.362	–	**12.7** / **0.458**
9BZ1	2-way	89	Min	**7.54** / 0.458	11.78 / 0.326	–	12.07 / **0.503**	10.1 / 0.426
			Max	12.62 / 0.341	**11.78** / 0.326	–	25.78 / 0.305	20.4 / **0.359**
9BZC	2-way	89	Min	19.71 / 0.364	56.87 / 0.198	–	11.8 / **0.54**	**10.8** / 0.331
			Max	37.61 / 0.295	56.87 / 0.198	–	29.17 / 0.327	**24.5** / **0.377**
7YR6	2-way	176	Min	17.74 / 0.319	–	30.93 / 0.191	**10.07** / **0.44**	11.3 / 0.358
			Max	28.34 / 0.251	–	30.93 / 0.191	**14.98** / 0.306	18.6 / **0.339**
7YR7	2-way	176	Min	**15.89** / **0.378**	24.41 / 0.219	27.69 / 0.235	23.07 / 0.375	22 / 0.252
			Max	25.37 / 0.264	**24.41** / 0.219	27.69 / 0.235	32.98 / **0.273**	34.7 / 0.389
9C75	3-way	72	Min	19.82 / 0.316	25.97 / 0.235	–	**12.49** / **0.421**	14.5 / 0.355
			Max	21.18 / 0.244	25.97 / 0.235	–	**19.28** / 0.329	24.5 / **0.399**
9ELY	3-way	205	Min	55.8 / 0.177	29.19 / 0.325	–	**14.92** / **0.362**	23.2 / 0.280
			Max	97.82 / 0.1	29.19 / **0.325**	–	**25.74** / 0.216	32.2 / 0.214
9DCF	*k*-way	90	Min	–	74.59 / 0.142	–	62.0 / 0.293	**16** / **0.391**
			Max	–	74.59 / 0.142	–	78.16 / **0.288**	**23.1** / 0.220
8UYS	*k*-way	124	Min	**14.63** / **0.379**	58.7 / 0.072	33.08 / 0.333	62.68 / 0.135	17.4 / 0.333
			Max	33.78 / 0.223	58.7 / 0.072	33.08 / **0.333**	77.49 / 0.097	**24.7** / 0.269
8UO6	*k*-way	134	Min	**13.68** / **0.411**	25.46 / 0.251	14.34 / 0.299	18.62 / 0.358	17.6 / 0.367
			Max	28.08 / 0.255	25.46 / 0.251	**14.34** / 0.299	26.99 / 0.253	22.6 / **0.341**
8UYE	*k*-way	135	Min	21.21 / **0.387**	**14.04** / 0.351	25.08 / 0.333	58.89 / 0.106	17.9 / 0.257
			Max	28.9 / 0.312	**14.04** / **0.351**	25.08 / 0.333	76.55 / 0.083	24.3 / 0.222
8S95	*k*-way	157	Min	–	76.0 / 0.119	–	–	**18.8** / **0.295**
			Max	–	76.0 / 0.119	–	–	**26.9** / **0.326**
9CBU	*k*-way	387	Min	**31.95** / 0.222	56.54 / 0.105	–	33.68 / **0.236**	33.1 / 0.186
			Max	**41.86** / **0.162**	56.54 / 0.105	–	48.0 / 0.101	45.5 / 0.135
9J6Y	*k*-way	526	Min	202.9 / 0.072	53.43 / 0.131	–	**34.41** / **0.203**	42.8 / 0.152
			Max	258.92 / 0.037	53.43 / **0.131**	–	**50.26** / 0.096	55.8 / 0.106
9ISV	*k*-way	580	Min	–	–	–	–	**27.1** / **0.292**
			Max	–	–	–	–	**37.7** / **0.250**
9J3R	*k*-way	580	Min	–	–	–	–	**35** / **0.146**
			Max	–	–	–	–	**44.1** / **0.108**
8FZA	p-knot	30	Min	–	10.09 / 0.652	**2.0** / **0.858**	7.44 / 0.776	5.95 / 0.742
			Max	–	10.09 / 0.652	**3.21** / 0.767	8.94 / 0.775	8.33 / **0.813**
7QR3	p-knot	69	Min	9.23 / 0.482	14.01 / 0.389	–	9.44 / **0.673**	**9.01** / 0.364
			Max	**13.72** / 0.39	14.01 / 0.389	–	14.05 / 0.366	16 / **0.434**
7QR4	p-knot	69	Min	13.61 / 0.456	16.99 / 0.374	–	13.17 / **0.5**	**9.03** / 0.418
			Max	23.43 / 0.39	**16.99** / 0.374	–	20.91 / **0.438**	24.2 / 0.408

For 2-way molecules and pseudoknots, which are generally short in sequence length, SimRNA and iFoldRNA (Table 2) obtained competitive RMSD values. Performance differed across the test sets. In Test Set B, iFoldRNA and GARN2 ranked among the top-performing methods for 2-way molecules, while in Test Set A, several techniques achieved comparable results. Notably, although SimRNA showed limited performance for pseudoknot-containing structures in Test Set B, it performed better on Test Set A, indicating dataset-dependent variability. When ranking all template-free techniques by lowest RMSD achieved per molecule, GARN3 recorded 7 best results in Test Set B and 5 in Test Set A, compared to 4 and 1 for GARN2, respectively. Overall, these observations suggest a general advantage of GARN3 over GARN2 within the 2-way junction and pseudoknot categories, though performance differences vary across individual molecules.

For 3-way molecules, iFoldRNA demonstrated competitive performance, achieving low RMSD values in Test Set B. GARN3 also produced competitive results on Test Set A. However, SimRNA was unable to predict several molecules in this category, even when attempting to predict higher-order junctions. On Test Set A, SimRNA achieved better results for the molecules it could predict, suggesting that prediction difficulty may depend on the structural properties of the evaluated molecules. For 4-way or higher-order junctions (*k*-way), GARN3 achieved low RMSD values across the evaluated molecules, recording 3 lowest-RMSD results in Test Set B and 3 in Test Set A among template-free techniques. These results describe the distribution of performance within this category.

Among template-free techniques (Tables 1 and 2), SimRNA and iFoldRNA demonstrate competitive RMSD values for shorter molecules, whereas GARN3 achieves stronger results for higher-order junction structures and larger molecules.

For template-based modeling techniques, Table 3 and Table 4 report the simulation results for each tested molecule. Due to space constraints, MC-Sym and VFoldLA are not included in these summary tables. Their exclusion does not affect the interpretation of the results presented in this section, as neither method achieved the lowest RMSD for any of the evaluated molecules. The complete results for all template-based techniques, including MC-Sym and VFoldLA, are provided in S4 Table and S5 Table.

**Table 3:** Comparison with template-based modeling techniques using Test Set A. The highlighted values in each row aim to help the visualization of the top-ranked technique, considering the lowest RMSD and the highest TM-Score. The scores are independent, therefore the structure with best-ranked RMSD not necessarily is the one with best-ranked TM-Score.

Mol.	Type	Len.	RMSD /TM	FARFAR2	RNA-Composer	3dRNA	FebRNA	GARN3
1XHP	2-way	32	Min	**0.92** / **0.946**	1.93 / 0.800	1.35 / 0.880	1.41 / 0.876	3.1 / 0.698
			Max	2.58 / 0.702	**1.93** / **0.800**	9.37 / 0.369	2.88 / 0.656	5.83 / 0.578
1MNX	2-way	42	Min	2.17 / 0.754	**1.74** / **0.869**	1.91 / 0.821	2.21 / 0.757	3.29 / 0.592
			Max	8.64 / 0.389	**1.74** / **0.869**	8.05 / 0.441	2.52 / 0.711	4.63 / 0.499
1CQ5	2-way	43	Min	2.62 / 0.700	8.04 / 0.355	3.35 / 0.608	**0.9** / **0.938**	3.25 / 0.617
			Max	6.80 / 0.330	8.04 / 0.355	13.5 / 0.293	9.45 / 0.311	**6.26** / **0.530**
2RP0	2-way	27	Min	2.25 / 0.908	4.22 / 0.902	**0.48** / **0.984**	1.15 / 0.911	6.76 / 0.774
			Max	5.81 / 0.780	**4.22** / **0.902**	11.0 / 0.850	4.35 / 0.818	7.68 / 0.752
2N6S	2-way	36	Min	–	2.28 / 0.751	**1.35** / **0.874**	1.96 / 0.793	3.01 / 0.627
			Max	–	2.28 / 0.751	2.44 / 0.775	**2.13** / **0.778**	7.22 / 0.601
1Q29	3-way	41	Min	**3.47** / **0.595**	4.91 / 0.544	4.42 / 0.534	–	9 / 0.354
			Max	7.83 / 0.388	**4.91** / **0.544**	8.85 / 0.370	–	11 / 0.421
3DIR	3-way	174	Min	12.7 / 0.388	**0.6** / **0.988**	17.8 / 0.354	6.31 / 0.624	14.5 / 0.304
			Max	24.8 / 0.230	**0.6** / **0.988**	31.7 / 0.161	16.9 / 0.323	24.1 / 0.268
4P8Z	3-way	188	Min	15.7 / 0.343	26.7 / 0.210	–	**6.41** / **0.486**	18.6 / 0.380
			Max	29.1 / 0.251	26.7 / 0.210	–	**9.02** / **0.450**	27.5 / 0.234
3AM1	3-way	81	Min	10.7 / 0.510	1.23 / 0.913	**0.73** / **0.975**	–	12.9 / 0.350
			Max	18.8 / 0.363	**1.23** / 0.913	1.48 / **0.952**	–	16.7 / 0.342
4RZD	3-way	102	Min	**7.35** / **0.449**	12.5 / 0.411	21.1 / 0.383	–	10.1 / 0.376
			Max	**9.98** / 0.341	12.5 / **0.411**	64.2 / 0.202	–	16.4 / 0.377
4QKA	3-way	122	Min	12.3 / **0.406**	14.4 / 0.330	17.0 / 0.342	–	**11.2** / 0.382
			Max	17.3 / 0.309	**14.4** / 0.330	48.2 / 0.220	–	19.9 / **0.357**
1Z43	3-way	101	Min	8.23 / 0.570	**2.6** / **0.756**	11.9 / 0.430	–	11.8 / 0.348
			Max	29.6 / 0.311	**2.6** / **0.756**	30.4 / 0.292	–	22 / 0.319
4P9R	3-way	189	Min	27.6 / **0.403**	26.6 / 0.234	28.2 / 0.367	–	**19.9** / 0.297
			Max	32.4 / **0.245**	**26.6** / 0.234	91.6 / 0.043	–	33.9 / 0.237
4OQU	*k*-way	97	Min	9.71 / 0.444	18.4 / 0.378	**5.65** / **0.720**	–	11.6 / 0.348
			Max	18.9 / 0.319	18.4 / **0.378**	23.8 / 0.285	–	**18.2** / 0.277
4QK8	*k*-way	124	Min	10.8 / **0.398**	14.5 / 0.330	17.0 / 0.340	–	**9.43** / 0.344
			Max	17.3 / 0.322	**14.5** / 0.330	48.2 / 0.220	–	19.9 / **0.355**
5J01	*k*-way	418	Min	38.6 / 0.164	42.8 / **0.255**	39.5 / 0.199	–	**30.1** / 0.165
			Max	56.9 / 0.098	42.8 / **0.255**	87.6 / 0.059	–	**36.8** / 0.157
3J28	*k*-way	1533	Min	–	–	–	–	**53.2** / **0.054**
			Max	–	–	–	–	**68** / **0.056**
1C2W	*k*-way	2904	Min	–	–	–	–	**67.1** / **0.038**
			Max	–	–	–	–	**82.4** / **0.025**
2NBX	*k*-way	108	Min	–	11.9 / 0.405	**2.91** / **0.832**	20.1 / 0.423	16 / 0.303
			Max	–	**11.9** / **0.405**	21.3 / 0.320	24.7 / 0.334	20.8 / 0.369
2G1W	pseudo-knot	22	Min	1.60 / 0.893	2.56 / 0.833	**1.21** / **0.916**	–	5.46 / 0.834
			Max	3.38 / 0.729	**2.56** / **0.833**	14.7 / 0.785	–	7.59 / 0.810
1KAJ	pseudo-knot	32	Min	1.44 / 0.925	4.74 / 0.861	**1.24** / **0.934**	–	6.77 / 0.776
			Max	4.98 / 0.809	**4.74** / **0.861**	5.00 / 0.827	–	10.7 / 0.779
2ZUF	pseudo-knot	78	Min	11.6 / **0.406**	15.7 / 0.394	15.6 / 0.399	–	**6.6** / 0.359
			Max	18.4 / 0.339	15.7 / **0.394**	18.0 / 0.365	–	**8.88** / 0.391

**Table 4:** Comparison with template-based modeling techniques, using Test Set B. The highlighted values in each row aim to help the visualization of the top-ranked technique, considering lowest RMSD and highest TM-Score. The scores are independent, therefore the structure with best-ranked RMSD not necessarily is the one with best-ranked TM-Score.

Mol.	Type	Len.	RMSD /TM	FARFAR2	RNA-Composer	3dRNA	FebRNA	GARN3
8VQV	2-way	64	Min	4.59 / 0.621	–	4.73 / 0.654	**3.0** / **0.724**	8.05 / 0.418
			Max	12.94 / 0.38	–	10.57 / **0.443**	**9.12** / 0.424	11.3 / 0.362
8VVJ	2-way	64	Min	4.91 / **0.675**	10.31 / 0.443	**4.84** / 0.667	–	9.02 / 0.493
			Max	13.27 / 0.387	13.07 / 0.343	**10.61** / 0.446	–	12.7 / **0.458**
9BZ1	2-way	89	Min	**5.15** / **0.61**	6.57 / 0.567	18.0 / 0.436	–	10.1 / 0.426
			Max	14.79 / 0.341	**9.83** / **0.419**	25.62 / 0.31	–	20.4 / 0.359
9BZC	2-way	89	Min	**3.75** / **0.678**	6.47 / 0.538	18.25 / 0.438	–	10.8 / 0.331
			Max	15.95 / 0.319	**11.49** / **0.38**	25.3 / 0.326	–	24.5 / 0.377
7YR6	2-way	176	Min	19.39 / 0.347	24.15 / 0.288	28.29 / 0.313	–	**11.3** / **0.358**
			Max	28.04 / 0.202	37.41 / 0.188	107.11 / 0.115	–	**18.6** / **0.339**
7YR7	2-way	176	Min	19.8 / **0.327**	20.92 / 0.32	**19.3** / 0.32	–	22 / 0.252
			Max	**30.48** / 0.168	33.28 / **0.206**	44.02 / 0.157	–	34.7 / 0.389
9C75	3-way	72	Min	17.52 / **0.355**	–	**14.18** / 0.342	–	14.5 / **0.355**
			Max	19.47 / 0.225	–	**17.7** / 0.327	–	24.5 / **0.399**
9ELY	3-way	205	Min	29.06 / **0.354**	–	25.21 / 0.285	–	**23.2** / 0.280
			Max	54.88 / 0.15	–	36.13 / 0.159	–	**32.2** / **0.214**
9DCF	*k*-way	90	Min	–	–	**14.68** / **0.391**	–	16 / **0.391**
			Max	–	–	**22.15** / **0.304**	–	23.1 / 0.220
8UYS	*k*-way	124	Min	**10.45** / **0.477**	15.99 / 0.378	16.06 / 0.424	–	17.4 / 0.333
			Max	20.4 / 0.29	**17.32** / **0.322**	25.9 / 0.297	–	24.7 / 0.269
8UO6	*k*-way	134	Min	14.79 / 0.401	16.64 / 0.496	**10.18** / **0.57**	–	17.6 / 0.367
			Max	26.41 / 0.187	**17.94** / 0.424	26.09 / **0.442**	–	22.6 / 0.341
8UYE	*k*-way	135	Min	**8.11** / **0.539**	24.5 / 0.367	19.52 / 0.367	17.29 / 0.399	17.9 / 0.257
			Max	29.13 / 0.283	27.7 / 0.298	32.38 / 0.22	**20.31** / **0.337**	24.3 / 0.222
8S95	*k*-way	157	Min	–	–	**11.25** / **0.468**	–	18.8 / 0.295
			Max	–	–	**21.66** / **0.367**	–	26.9 / 0.326
9CBU	*k*-way	387	Min	37.93 / **0.287**	–	39.35 / 0.157	–	**33.1** / 0.186
			Max	59.63 / 0.127	–	104.97 / 0.041	–	**45.5** / **0.135**
9J6Y	*k*-way	526	Min	–	–	–	–	**42.8** / **0.152**
			Max	–	–	–	–	**55.8** / **0.106**
9ISV	*k*-way	580	Min	–	–	–	–	**27.1** / **0.292**
			Max	–	–	–	–	**37.7** / **0.250**
9J3R	*k*-way	580	Min	–	–	–	–	**35** / **0.146**
			Max	–	–	–	–	**44.1** / **0.108**
8FZA	p-knot	30	Min	–	9.94 / **0.819**	**3.25** / 0.718	–	5.95 / 0.742
			Max	–	11.4 / 0.62	**7.3** / 0.654	–	8.33 / **0.813**
7QR3	p-knot	69	Min	11.44 / 0.457	18.72 / 0.331	13.16 / **0.46**	–	**9.01** / 0.364
			Max	16.23 / 0.329	18.72 / 0.331	36.83 / 0.364	–	**16** / **0.434**
7QR4	p-knot	69	Min	12.51 / **0.452**	17.1 / 0.39	12.18 / 0.443	–	**9.03** / 0.418
			Max	**15.39** / 0.313	18.53 / 0.329	25.98 / 0.34	–	24.2 / **0.408**

For template-based approaches applied to molecules with 2-way junctions, 3dRNA and FebRNA achieved competitive RMSD values across most evaluated cases. These methods remained among the strongest performers for this category in both evaluation settings.

For 3-way and higher-order (*k*-way) junctions, FARFAR2 obtained low RMSD values in several cases. In molecules with *k*-way junctions, GARN3 also achieved competitive results. Because some techniques have length limitations, molecules such as 3J28 and 1C2W were predicted only by GARN3.

Among template-based techniques, 3dRNA and FebRNA consistently demonstrated to achieve low RMSD values for 2-way junctions, while FARFAR2 and GARN3 are the stronger performers for 3-way and higher-order junctions, particularly for larger molecules.

When comparing results across the two evaluation sets, GARN3 achieved the most lowest-RMSD results in both settings: 14 in Table 3 and 8 in Table 4, compared to FARFAR2 (10 and 4) and 3dRNA (8 and 7). These results describe the distribution of performance across evaluation conditions, particularly for structurally complex molecules.

When evaluating the results of deep-learning modeling techniques (see S6 Table and S7 Table), trRosettaRNA achieved the lowest RMSD values for the majority of molecules (14 out of 22). This trend was observed consistently across the two evaluation sets: trRosettaRNA achieved 12 wins in Test Set B and 14 in Test Set A, highlighting its strong generalization capability. AlphaFold 3 also demonstrated competitive results, followed by GARN3. AlphaFold 3 recorded 5 lowest-RMSD results in Test Set A and 2 in Test Set B, while GARN3 recorded 3 and 6, respectively. These values describe the distribution of performance across methods within this modeling category.

When the results from GARN2 and GARN3 are compared, a relevant difference is observed. In summary, 13 of the 22 tested molecules from Test Set A achieved lower RMSD values with GARN3 (Tables 1 and 3). Among the molecules better predicted by GARN2, no clear pattern is apparent other than their relatively small structural length. When the TM-score is considered instead of RMSD, the advantage of GARN3 is even more pronounced: on Test Set A, GARN3 achieved a higher maximum TM-score than GARN2 for 14 of the 20 molecules where both produced valid results, including 5 cases where GARN3’s minimum RMSD was slightly higher. This suggests that GARN3 tends to explore conformations with better overall fold quality even when individual distance metrics are marginally worse. The advantage was particularly consistent for *k*-way junctions, where GARN3 achieved lower RMSD values than GARN2 in 5 out of 6 cases, and for the three largest molecules in Test Set A (ranging from 418 to 2904 nucleotides), where GARN3 produced lower RMSD values in all three cases. Additionally, GARN3 produced valid predictions for all evaluated molecules, whereas GARN2 results were not available for 2 molecules in Test Set A and 5 in Test Set B. A similar trend is observed on Test Set B (Table 2), where GARN3 recorded 7 lowest-RMSD results among template-free techniques, compared to 4 for GARN2, and achieved higher TM-scores in 12 of the 15 molecules where both techniques produced results.

### 5.3. Classification of best molecules

In our simulations, most molecules with 2-way junctions achieved better simulation results when larger maximum-distance thresholds were considered. These molecules are generally short, with fewer than 100 nucleotides. The only exception was molecule 2RP0, which achieved better results with the lowest maximum distance; although classified as a 2-way topology, it also contains a pseudoknot. Likewise, among molecules with pseudoknots, most yielded better simulation results with lower maximum-distance thresholds. This observation is consistent with the more constrained conformational space associated with pseudoknot interactions, as also reflected in the distance–RMSD trends.

For molecules with 3-way junctions, the opposite trend was observed: classification accuracy improved with lower maximum-distance thresholds. This effect became increasingly pronounced in larger molecules, particularly in helices with a greater number of nucleotides. As shown in S4 Fig and S5 Fig, greater structural length and complexity appear to amplify the benefit of stricter distance constraints: smaller maximum distances guide the sampling process toward structurally more accurate conformations, as evidenced by the lower RMSDs achieved. This behavior manifests as the downward trends observed across several of the 3-way junction molecules.

For molecules with *k*-way junctions, the pattern of smaller distances is also visible. One of the molecules with fewer nucleotides, 2NBX, exhibits the opposite behavior, possibly due to its smaller size compared to the other *k*-way molecules. This behavior is also apparent in the scatter plots shown in S6 Fig and S7 Fig: for 2NBX, the correlation between maximum distance and RMSD is weaker or inverted compared to other *k*-way molecules, which may reflect a possible influence of molecular size on the effect of the maximum-distance parameter.

When considering Test Set B, the same overall tendencies remain visible: molecules with simpler 2-way junctions generally favor higher maximum distances, whereas those with 3-way or *k*-way topologies perform better with smaller ones. This correspondence between the two evaluation sets is consistent with a junction-dependent effect of the sampling radius.

Based on the observed trends, UCB may be suitable for molecules with at most 2-way junctions, whereas EXP3 may be appropriate for molecules with 3-way or higher-order junctions; when feasible, evaluating both algorithms can help assess robustness. All results are reported in S4 Fig, S6 Fig, and, for Test Set B, in S5 Fig and S7 Fig.

### 5.4. Execution time

Another important aspect to evaluate is the execution time, to determine whether GARN3 maintains optimized prediction performance compared to other techniques in the literature, such as GARN/GARN2 [10,17]. The GARN2 model uses a smaller number of players than the GARN3 model, particularly for larger molecules with extended helices. Therefore, it is expected that the simulation time of GARN3 will increase compared to GARN2, while maintaining competitive time efficiency relative to other techniques.

These expectations are supported by the execution times reported for Test Set A in S10 Table and for Test Set B in S11 Table. For molecules listed in S10 Table, GARN3 completes simulations for small and medium-sized molecules within minutes and scales to a few hours for very large structures. For example, 3J28 (1,533 nt) required approximately 2 h 51 min, and 1C2W (2,904 nt) approximately 3 h 18 min, despite the increased number of pseudoatoms compared to GARN2. Across the remaining test cases, GARN3 typically completes simulations in approximately 1–15 minutes for molecules under approximately 200 nucleotides (e.g., 4QKA, 4OQU, 4QK8), illustrating a consistent scaling behavior from small to larger molecules.

The execution times observed for Test Set B further support the efficiency of GARN3. In many cases, simulations completed within seconds (e.g., 7QR3 ∼2s, 7QR4 ∼2s, 9DCF ∼7s, 9C75 ∼7s), and remained under one minute for most other targets (e.g., 8UYE ∼31s, 8UYS ∼20s). These times are substantially shorter than those reported for web-based tools, which often require tens of minutes to several hours.

Compared with several alternative methods, GARN3 exhibits shorter execution times than MC-Sym, trRosettaRNA, 3dRNA, SimRNA, and FARFAR/FARFAR2, which may take several hours to days for large molecules. A limited number of techniques show comparable or lower execution times, including AlphaFold 3, RNAComposer, FebRNA, NAST, and GARN2. In the case of GARN2, its shorter execution time is primarily due to its reduced number of players in the representation rather than differences in predictive accuracy.

Comparing execution times between web server-based techniques (such as AlphaFold 3, trRosettaRNA, and MC-Sym) and local execution is not entirely fair. Web server times include factors such as server queues and network delays, not just the algorithm’s performance. Additionally, each technique can be hosted in different cloud servers, using different machines or setups. Timing measurements, therefore, do not necessarily reflect the true computational efficiency of each method. With this perspective, what stands out is the difference between GARN3, GARN2, and other techniques we could run locally on the same machine configuration.

Overall, GARN3 maintains competitive execution-time performance. Although it is slightly slower than GARN2 due to its richer pseudoatomic representation, it remains faster than most existing techniques and scales effectively to very large RNA molecules.

### 5.5. Aggregate view across techniques and test sets

**Fig 11:**
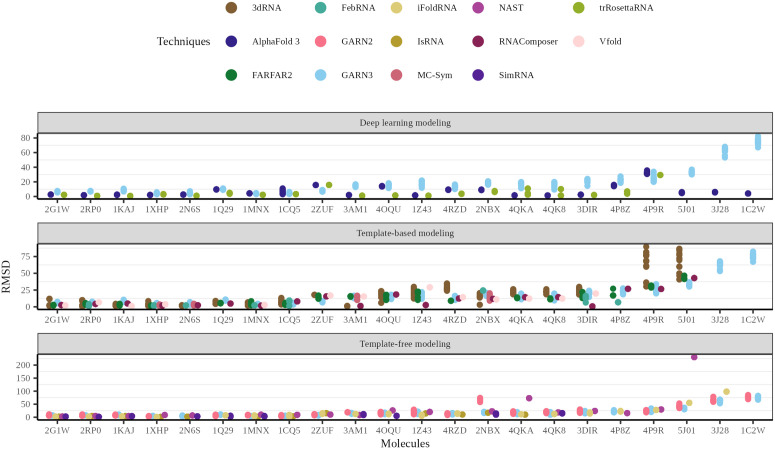
Comparison of RMSD evaluation with other techniques, considering Test Set A, where the results are split by the technique’s simulation type (template-based, template-free, and deep learning). GARN3 is present in the 3 categories, only to compare with the other techniques. The result from GARN3 in this graph is the best-performing sampling method across both EXP3 and UCB algorithms.

**Fig 12:**
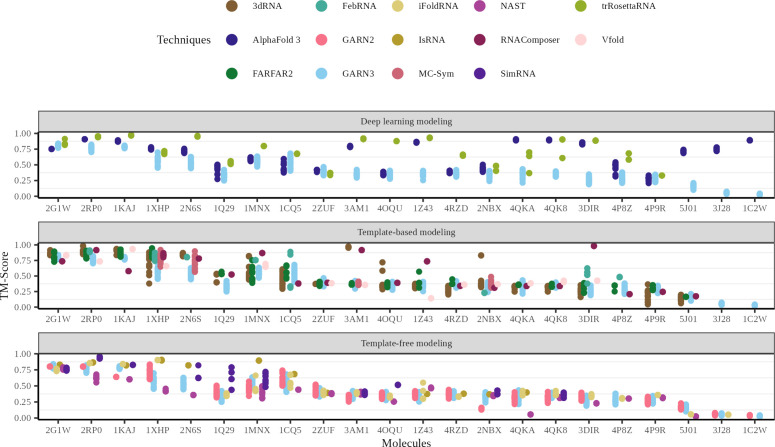
Comparison of TM-Score evaluation with other techniques, considering Test Set A, where the results are split by the technique’s simulation type (template-based, template-free, and deep learning). GARN3 is present in the 3 categories, only to compare with the other techniques. The result from GARN3 in this graph is the best-performing sampling method across both EXP3 and UCB algorithms.

**Fig 13:**
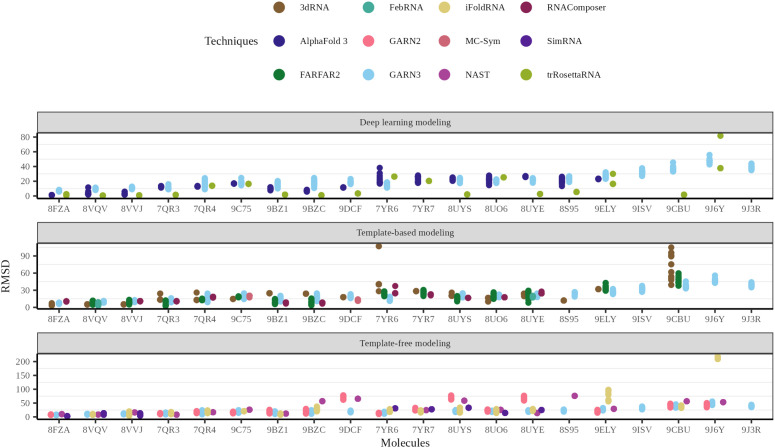
Comparison of RMSD evaluation with other techniques, considering Test Set B, where the results are split by the technique’s simulation type (template-based, template-free, and deep learning). GARN3 is present in the 3 categories, only to compare with the other techniques. The result from GARN3 in this graph is the best-performing sampling method across both EXP3 and UCB algorithms.

**Fig 14:**
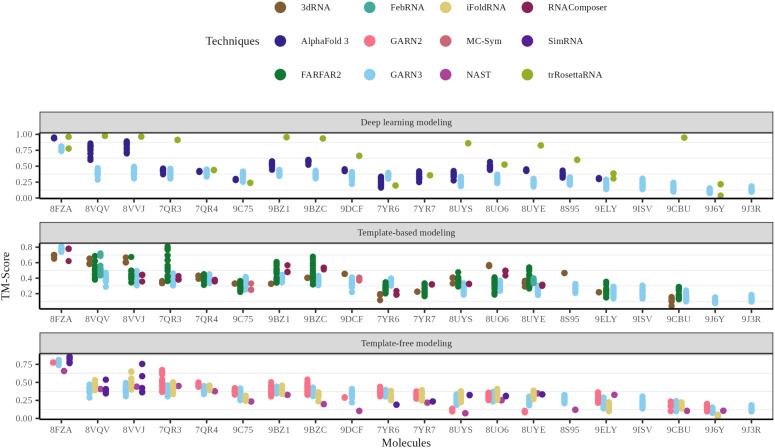
Comparison of TM-Score evaluation with other techniques, considering Test Set B, where the results are split by the technique’s simulation type (template-based, template-free, and deep learning). GARN3 is present in the 3 categories, only to compare with the other techniques. The result from GARN3 in this graph is the best-performing sampling method across both EXP3 and UCB algorithms.

To complement the per-molecule tables (Tables 1 to 4), we provide in Figs 11–14 an aggregate view of the prediction results across the three categories of techniques considered in this study (template-based, template-free, and deep learning), and across the two test sets. Each figure groups the methods by category and reports either RMSD or TM-score on a per-molecule basis, with GARN3 shown in each category to enable a direct visual comparison with the other techniques in that category. For each figure, the GARN3 entry corresponds to the best-performing sampling method across the EXP3 and UCB algorithms.

For Test Set A, Fig 11 reports RMSD and Fig 12 reports TM-score. For Test Set B (CASP targets), Fig 13 and Fig 14 report the analogous quantities. The set of molecules and methods is identical to that of the per-molecule tables of the preceding subsections. The interpretation of these aggregate views and their comparison across categories and test sets is provided in Section 6.

## 6. Discussion

The main objective of this work is to improve the GARN2 technique by introducing a finer representation of helices through the addition of more players, thereby enhancing the visualization of the predicted molecules in the proposed technique, GARN3. To achieve this improvement, we updated the game strategies and introduced a revised scoring procedure based on a machine learning regression model.

Simulations were then performed on Test Sets A and B, comparing GARN3 with GARN2 and with the other techniques reported in the literature. On Test Set A, GARN3 achieved lower RMSD values than GARN2 for 13 out of 22 molecules; on Test Set B, GARN3 obtained 7 of the lowest-RMSD results among template-free methods, compared to 4 for GARN2, with the remaining targets showing values comparable within the variability of the evaluation. This comparison is not strictly equivalent: the GARN2 and GARN3 representations differ in granularity, so RMSD is evaluated over different sets of pseudoatoms, and the observed differences reflect the combined contribution of the refined helix representation, the updated strategy set, and the regression-based scoring function rather than any single isolated factor. These results therefore indicate that GARN3 produces a more detailed coarse-grained model with at least comparable accuracy. The contribution of GARN3 is in this sense methodological: a finer representation and a learned scoring function made tractable within the GARN framework, rather than a quantitative improvement in predictive accuracy over GARN2.

One point of attention after updating the game configuration, in addition to the refinement of the 3D model representation, was the execution time, as this has been a key advantage of GARN/GARN2 compared with other techniques. In our simulations, GARN3 showed an increase in simulation time as the molecules grew, typically exceeding 100 nucleotides, as expected given the increase in players. However, the technique remained comparable to, and in many cases faster than, other methods in terms of simulation time, and structures containing several hundred nucleotides remained tractable on a local machine (see S10 Table and S11 Table for details on the simulation times).

The aggregate view of Test Set A (Fig 11) shows that GARN3 achieves RMSD values equivalent to or lower than those of several other techniques for a substantial fraction of molecules. Compared with template-based techniques, the differences are most visible on molecules with 3-way junctions and on *k*-way junctions of fewer than 200 nucleotides. Compared with deep learning-based techniques, GARN3 yields results similar to AlphaFold 3, while trRosettaRNA achieves lower RMSD values for most molecules, particularly for larger or highly branched structures.

The TM-score view of Test Set A (Fig 12) is consistent with the trends observed in the RMSD analysis: GARN3 obtains mid-to-high TM-scores across many targets, indicative of reasonable global topology reconstruction in several cases. As with RMSD, trRosettaRNA frequently attains higher TM-scores, while GARN3 yields values comparable to those of AlphaFold 3 and, for some molecules, to several template-free methods. For molecules in which GARN3 achieves lower RMSD values, the corresponding TM-scores generally fall within a moderate-to-high range, which may reflect agreement not only at a local level but also at the level of global fold similarity.

On Test Set B (Figs 13 and 14), GARN3 performs comparably to several template-based methods. In contrast, deep learning approaches (AlphaFold 3 and trRosettaRNA) achieve lower RMSD and higher TM-score values for most targets, particularly for simpler 2-way junctions and many *k*-way structures. GARN3 appears to perform comparatively better on larger, more structurally complex molecules (> 500 nt), where some deep learning methods may be unavailable or less applicable. These observations suggest that GARN3 may serve a complementary role rather than directly matching the overall predictive accuracy of AI-based approaches.

However, it is important to note that the stronger performance of trRosettaRNA and AlphaFold 3, as measured by RMSD and TM-score, relative to GARN3 should be interpreted in light of differences in the training data. The trRosettaRNA model was trained using PDB structures released before January 2022 [36], with additional filtering steps described in its methodology, and AlphaFold 3 included experimentally determined structures available before September 30, 2021 [3]. Because the molecules in Test Set A were deposited before these dates, partial overlap between the evaluation set and the training data for these deep learning models cannot be ruled out. This does not imply direct data leakage; however, it indicates that their performance on Test Set A may reflect prior exposure to related structural patterns. In contrast, GARN3 was evaluated on structures that were not included in its regression model training.

Regarding the scoring function, the dynamic distances predicted by the machine learning regression model reduce the need to manually specify parameters that depend on the SSE type, thereby improving the approach’s flexibility. As noted previously, in some cases, the learned scoring function enables predictions for molecules that could not be obtained using static parameters. This effect is reflected in the corresponding RMSD and TM-score values for several targets.

In the methodology section, the maximum distance in the structure is described as the sorting strategy for selecting the best predictions. This criterion, which is used and validated in GARN2, brought good results to the GARN3 simulations. In several molecules, the best results are close to the maximum distance, whereas others are close to the minimum.

**Fig 15:**
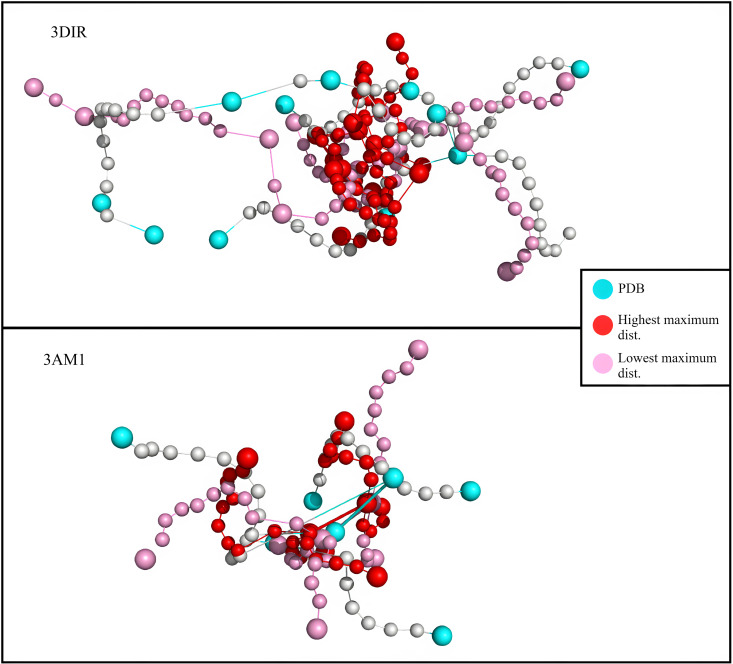
Sorting strategy used in the molecules. **The native structure is compared to both the maximum and minimum distances.** In our simulations, 3DIR has one of the best-evaluated structures with the highest maximum distance, whereas 3AM1 is the opposite, with one of the best samplings from the lowest maximum distance.

In our simulations, the best-performing simulations with the lowest maximum distances are mainly observed in molecules with pseudoknots and in those with 3-way or higher-order junctions. Another pattern observed was that the best simulations had the highest maximum distances, mostly in smaller molecules with 2-way junctions. An example is shown in Fig 15, where molecule 3DIR exhibits one of the best-evaluated structures with the highest maximum distance, while 3AM1 shows one of the best results at a lower maximum distance. Similar junction-dependent trends are also visible in the evaluation of molecules from Test Set B (see Fig 7), suggesting that the preferred distance scale varies with topological complexity.

A limitation not explored here concerns the combination of global and local scores, since GARN2 and GARN3 compute scores locally at each node. This is why we decided to use the previously validated sorting criterion in GARN2, which produced similar results in this study. Another limitation that remains is the number of pseudoatoms (players) in the GARN representation. The technique presents a coarse-grained model that may be outdated compared to newer techniques that produce near-native 3D models. During development, we also evaluated a one-bead-per-nucleotide representation (one player per nucleotide) across the whole structure; although this representation is more uniform, it became computationally expensive and less accurate, because the number of strategies grows quickly with each additional player under regret-minimization dynamics. This trade-off is why we adopted the current intermediate representation, which balances structural detail and computational tractability (see S2 Appendix for more details). In this context, GARN3, like GARN and GARN2, runs on local machines, remains computationally efficient (as discussed in the execution-time analysis), does not require external library dependencies, and does not require a dedicated GPU (Graphic Processing Unit). Future work may also investigate hybrid global–local scoring strategies, as well as adaptive distance schedules, to further improve RMSD and TM-score performance while preserving computational efficiency.

### 6.1. Intended use cases and positioning of GARN3

The benchmarking results suggest that GARN3 is best positioned as a complementary tool rather than a direct competitor of state-of-the-art deep learning predictors. Three contexts in particular favor its use:

1. **Large RNA molecules.** For structures exceeding roughly 500 nucleotides, several deep learning methods become impractical due to memory requirements, sequence-length limits of public web servers, or the absence of multiple sequence alignments of sufficient depth. GARN3 produced predictions for all targets in our test sets, including molecules of 1 533 and 2 904 nucleotides for which several other tools returned no result.
2. **Coarse-grained exploratory analysis.** When the objective is to obtain a global topology of the molecule, evaluate alternative folding hypotheses, or generate ensembles of conformations consistent with a given secondary structure, GARN3 provides interpretable, reproducible predictions at low computational cost.
3. **Settings without GPU access or external dependencies.** GARN3 runs locally on a standard CPU, requires no training data beyond what is distributed with the source code, and has no dependency on external services or proprietary models. This makes it suitable for educational use, for pipelines with strict data-locality constraints, and for users who need a self-contained baseline.

We do not claim that GARN3 matches the local accuracy of methods such as AlphaFold 3 or trRosettaRNA on targets where these methods are applicable. Rather, the contribution is methodological: GARN3 demonstrates that a game-theoretic, regret-minimization sampler combined with a learned scoring function can produce RNA models with consistent global topology across a wide range of sizes and junction complexities, without relying on large databases of homologous structures.

## 7. Conclusion

The results presented in this study introduce GARN3, a coarse-grained approach that achieves accuracy comparable to GARN2 on the evaluated benchmarks while providing a substantially finer 3D representation, and that remains competitive with several other template-free techniques. We improved the scoring function used in GARN2 by incorporating a machine learning regression model to estimate its parameters, making the scoring process more reliable since we no longer need to manually calculate them, as in previous versions of GARN [10,17]. The final 3D structure generated by GARN3 contains more pseudoatoms than GARN2 (see S12 Table and S13 Table), providing a finer approximation of the native structure. The simulations conducted on the test set in this study showed that GARN3 produced results similar to those of other template-free modeling techniques, while also remaining competitive with deep learning methods such as AlphaFold 3 in several molecules. In addition, the TM-score analysis indicated that many of the predicted structures preserve the correct global fold, complementing the improvements observed in RMSD.

In addition, GARN3 maintains the efficiency in large-molecule simulations, similar to GARN2, although scalability remains a challenge for many recent techniques.

During our tests, GARN3 demonstrates competitive performance across diverse molecular sizes and topologies, as a coarse-grained approach that relies on no large-scale experimental structure databases for training. In contrast, while deep learning methods (AlphaFold 3 and trRosettaRNA) achieve lower RMSD values, their superior performance may be partially attributable to exposure during training to structures released before the evaluation date; both methods include PDB data deposited before their respective cutoff dates (trRosettaRNA: before January 2022; AlphaFold 3: before September 30, 2021), which overlaps with Test Set A. GARN3’s junction-optimization strategy and scalability to very large molecules (> 1500 nt) position it as a valuable complementary approach, particularly where training-data independence and local execution are important.

Furthermore, GARN3 maintains practical execution time efficiency across all evaluated targets. The simulations completed in seconds to minutes for most molecules under 200 nucleotides and scaled to hours even for very large structures exceeding 1500 nucleotides, demonstrating that GARN3 preserves the computational advantage that characterized earlier GARN versions. This efficiency, combined with the ability to run locally without external dependencies or GPU requirements, makes GARN3 an accessible and interpretable alternative to web-based or computationally intensive approaches.

Overall, GARN3 combines increased structural resolution with a machine-learning-based scoring function and demonstrates competitive predictive performance across the evaluated benchmarks. The method remains computationally efficient and can be executed locally, which may be relevant in contexts where resource constraints and interpretability are important considerations.

The implementation of the GARN3 technique is available at https://github.com/jhonatans01/garn3, written and executable in Java. The repository contains the GARN3 file executable by any Java Virtual Machine (Java 17 or higher), along with a list of instructions for all available functions and parameters.

## Supporting information

S1 Appendix Molecules used in the analysis. All molecule IDs that were used for analysis, both to create the knowledge-based potential and to generate the machine learning prediction model.
(PDF)

S2 Appendix Machine Learning Model using a GARN model with 1-bead representation. This section contains the evaluation previously done for the 1-bead representation in GARN, related to the scoring function update.
(PDF)

S1 Fig Example of GARN3 graph representation. The players in blue color represent helix pseudoatoms (base-pairs). The players in yellow represent pseudoatoms in terminal loops, 2-way junctions, and 3-way junctions. Finally, the players in purple and green colors represent *k*-way (*k* > 3) junction pseudoatoms.
(PDF)

S2 Fig GARN players ordered. The players of the highest-order junction (in the example, it is a 3-way junction) play first, and the order of the other players is defined by applying a depth-first search algorithm, starting the search from the first highest-order junction.
(PDF)

S3 Fig Best simulations for molecule 1XHP. This figure presents the best simulation for molecule 1XHP, using each of the techniques tested here. SimRNA is absent because it cannot be generated. GARN2 is also absent, as it uses a different model with fewer pseudoatoms.
(PDF)

S4 Fig Maximum distance of GARN3 sampling for Test Set A. This plot presents the highest maximum and lowest maximum distances between the nodes, for each of the molecules in Test Set A.
(PDF)

S5 Fig Maximum distance of GARN3 sampling for Test Set B. This plot presents the highest maximum and lowest maximum distances between the nodes, for each of the molecules in Test Set B.
(PDF)

S6 Fig Relation between RMSD and the maximum distance of GARN3 sampling, for Test Set A. This plot presents the RMSD and the maximum distances between the nodes for each of the molecules in Test Set A.
(PDF)

S7 Fig Relation between RMSD and the maximum distance of GARN3 sampling, for Test Set B. This plot presents the RMSD and the maximum distances between the nodes for each of the molecules in Test Set B.
(PDF)

S1 Table Test Set A, using molecules from RNA FRABASE. Molecules used to run the simulations and perform the evaluation. This test set contains 22 molecules, where primary and secondary structures were obtained from the RNA FRABASE repository.
(PDF)

S2 Table Test Set B, using molecules from CASP (Critical Assessment of Structure Prediction) 15 and 16. Molecules used to run the simulations and perform the evaluation. This test set contains 20 molecules, and it considers only RNA structures evaluated in CASP 15 and 16. Each target ID is referenced with its corresponding PDB ID and description.
(PDF)

S3 Table Algorithms tested using the dataset of molecules from this study, excluding all molecules used in our predictions and evaluation. The columns represent the R^2^, MAE (Mean Absolute Error), and RMSE (Root Mean Squared Error) scores obtained after cross-validation. The values are sorted by R^2^ score, in descending order.
(PDF)

S4 Table Template-based techniques simulation results from Test Set A. Comparison of GARN3 with other techniques, considering only template-based techniques. The scores are independent, therefore the structure with best-ranked RMSD not necessarily is the one with best-ranked TM-Score.
(PDF)

S5 Table Template-based techniques simulation results from Test Set B. Comparison of GARN3 with other techniques, considering only template-based techniques. The scores are independent, therefore the structure with best-ranked RMSD not necessarily is the one with best-ranked TM-Score.
(PDF)

S6 Table Deep learning-based techniques’ simulation results from Test Set A. Comparison of GARN3 with other techniques, considering only techniques based on a deep learning approach. The scores are independent, therefore the structure with best-ranked RMSD not necessarily is the one with best-ranked TM-Score.
(PDF)

S7 Table Simulation results for deep learning-based techniques on Test Set B. Comparison of GARN3 with other techniques, considering only techniques based on a deep learning approach. The scores are independent, therefore the structure with best-ranked RMSD not necessarily is the one with best-ranked TM-Score.
(PDF)

S8 Table Simulations using both regret minimization algorithms in Test Set A. Comparison of GARN3 simulations using the UCB and EXP3 algorithms.
(PDF)

S9 Table Simulations using both regret minimization algorithms in Test Set B. Comparison of GARN3 simulations using the UCB and EXP3 algorithms.
(PDF)

S10 Table Simulation time of the molecules from Test Set A. The asterisk (*) next to the technique name indicates that the simulations were run on a local machine, while the others were run on its dedicated web server. The VFoldLA is not present because, even when providing different email addresses from different domains, the web server did not send any notification when the simulations finished.
(PDF)

S11 Table Simulation time of the molecules from Test Set B. The asterisk (*) next to the technique name indicates that the simulations were run on a local machine, while the others were run on its dedicated web server. The VFoldLA is not present because, even when providing different email addresses from different domains, the web server did not send any notification when the simulations finished.
(PDF)

S12 Table Players in GARN2 and GARN3 models for Test Set A. Quantity of players used when simulating the molecules in Test Set A, considering GARN2 and GARN3 models.
(PDF)

S13 Table Players in GARN2 and GARN3 models for Test Set B. Quantity of players used when simulating the molecules in Test Set B, considering GARN2 and GARN3 models. The empty values in GARN2 indicate that the technique returned errors when the molecules were used as input.
(PDF)
