# Supplementary material for: GARN3: A coarse-grained helix centered technique for RNA 3D structures prediction
Source: PLoS One. 2026 Jun 22;21(6):e0328609. doi: 10.1371/journal.pone.0328609 (PMC13286185; doi:10.1371/journal.pone.0328609)
Supplement: S1 Appendix — All molecule IDs that were used for analysis, both to create the knowledge-based potential and to generate the machine learning prediction model. (PDF) [file pone.0328609.s001.pdf]

S1 Appendix - Molecules used for analysis / machine learning model

The following PDB ids were used both for analysis to create the knowledge-based potential and to generate the machine learning prediction model: 3IAB, 3P59, 1VBX, 1ATO, 1J1U, 1QRS, 1AJU, 2DU4, 2MQT, 1ANR, 1AQO, 2RPT, 1U0B, 3MUT, 3F2Q, 2B7G, 2LA9, 1EUY, 2LV0, 1K6G, 2ES5, 1ARJ, 3B31, 4R4V, 3IYR, 1VBY, 1B36, 2MIS, 1A4D, 1QF6, 3GOG, 2MER, 1DDY, 2ZH9, 2F88, 1OW9, 3E5C, 1DUQ, 1EHT, 1EI2, 1RNK, 1K5I, 1ET4, 3D2G, 1EBR, 3D0X, 4FEO, 2RD2, 4FEN, 3MUV, 4QLN, 1EVV, 2KDQ, 1B23, 4CQN, 1EBS, 4FRG, 1F7F, 3SKT, 1ZO1, 2DU6, 3IYQ, 2O3V, 1SJ4, 1FFY, 2ZH8, 1VBZ, 1MWL, 1F27, 3GAO, 2M21, 1DRZ, 2M1O, 1FIR, 4GMA, 1A4T, 1A51, 1FEQ, 1SJF, 2F8K, 2OEU, 3IZY, 1QRU, 1T4L, 1F7G, 1F7H, 3E5F, 2HOP, 1Y27, 2LBS, 3F2W, 3MUR, 4FEJ, 2B63, 3CW5, 2LBR, 1Y26, 3DD2, 1F7I, 1F84, 3MOJ, 2ESE, 1L2X, 1F85, 4R4P, 429D, 1FOQ, 3L0U, 1QRT, 2DU3, 2DVI, 2O32, 1QFQ, 1TXS, 4WFL, 28SR, 3GOT, 1SJ3, 2AKE, 1HLX, 2OIU, 3E5E, 1E95, 2G1G, 1U3K, 3D2V, 3F2T, 2B6G, 3F30, 2LBQ, 3CW6, 2L6I, 1NEM, 3NVI, 1HR2, 3DHS, 1L1W, 1EOR, 1IE2, 2JWV, 2OE6, 1VTQ, 3LQX, 1IKD, 2FY1, 3GCA, 1DUL, 3BWP, 1Q8N, 2M5U, 1SY4, 1SZY, 3BNR, 2A9L, 357D, 2QH2, 1SCL, 1JO7, 2XNZ, 2O81, 3IRW, 2GOZ, 3CJZ, 3MJB, 3DW7, 1K9W, 1L8V, 1LDZ, 480D, 3OK2, 1M5L, 1M82, 1N66, 2L3C, 3DW6, 3ZD3, 2PN3, 3EGZ, 1ZIG, 2A64, 2QH3, 1NBR, 2DR2, 1OSW, 3BNS, 1QTQ, 2HGH, 1P5M, 1P5N, 1O0B, 1F79, 3GER, 4WCP, 2MXL, 422D, 1P5O, 3B4B, 1Q93, 1Q96, 2EVY, 2N2P, 3MJA, 2K4C, 1BYJ, 1YMO, 1LVJ, 2KOC, 2PXK, 1QC8, 3F4G, 1ESY, 3DW5, 1R2P, 2G5K, 1WTT, 3IQN, 1XSH, 2O83, 1JOX, 1MFQ, 1J7T, 1Q75, 3GES, 1TRA, 1O0C, 5DI2, 5DH6, 1CSL, 1O15, 1AFX, 2QH4, 3LA5, 2DR5, 1R7W, 1QU3, 2ZY6, 1R7Z, 1NYB, 2YDH, 2RO2, 3A3A, 1S9S, 1Y0Q, 2K96, 1LUU, 3FO4, 462D, 2KTZ, 3MJ3, 2KX8, 1T28, 2ZZM, 2DRB, 1QU2, 1YLG, 1F6X, 1YNE, 2AU4, 2FQN, 1F6Z, 2FK6, 3K1V, 2MXJ, 4TZX, 1ZC5, 1ZCI, 1RFR, 1NBK, 2R8S, 1UUU, 3FO6, 1ZO3, 2PXL, 1N8X, 2NQP, 2LI4, 2K95, 2D1B, 2GCV, 2KXM, 2KXZ, 2GDI, 2H0W, 2H0X, 2H0Z, 4YB0, 1H4Q, 1HOQ, 4KQY, 4NYA, 1QWB, 4B5R, 2IL9, 1XST, 2IXY, 3IQR, 3FU4, 2KPV, 2G9C, 1KH6, 2K5Z, 2PXV, 4FAX, 2K66, 2L3J, 3DVZ, 1LNG, 2KU0, 1LC6, 2KRL, 2KUR, 1RHT, 1XSU, 1TFN, 2KUV, 1TJZ, 3GS5, 3IGI, 2KZL, 2DR9, 1QWA, 4NYB, 2L1F, 1ZJW, 2KX5, 2LDT, 1U9S, 2GCS, 1LUX, 2PXT, 2PXU, 3Q51, 4A4S, 2LKR, 2LK3, 2PXB, 3SUX, 2LPS, 2N2O, 1EHZ, 2KY0, 3IQP, 2JSE, 4NYC, 2X7N, 2DR8, 2MHI, 1F9L, 2FRL, 1Q9A, 1H4S, 4PHY, 2MIY, 1A3M, 2DET, 1SYZ, 1MFK, 1ME1, 1HWQ, 4R0D, 2MTK, 2N1Q, 1NZ1, 2EUY, 2YIF, 3CGR, 1PJY, 2PXQ, 2PXF, 2PXP, 1KP7, 1U8D, 2N3Q, 2AB4, 1ZIH, 2H0S, 1ME0, 1F7U, 5C7U, 2M8K, 4LX6, 5DH8, 2N3R, 2M58, 5C7W, 2CD1, 4NYD, 2HEM, 1SLO, 2PCV, 2QUS, 2QUW, 1EIY, 4MEH, 2YIE, 2KVN, 2QWY, 2RLU, 3WFS, 2BE0, 2PXE, 2PXD, 3F4H, 4FB0, 2L2K, 1L9A, 1NUJ, 3B4C, 1XSG, 2JRG, 2H2X, 3D2X, 397D, 1F7V, 1XJR, 3DS7, 2ZH3, 2HW8, 1TN1, 2QBZ, 3F4E, 2GIP, 3FU2, 3SD1, 3OWI, 3OWZ, 2LUB, 3OWW, 1EUQ, 3OX0, 1YG3, 3F2X, 4TZY, 3F2Y, 2LC8, 1YSV, 3OXB, 2RPK, 2PJP, 3OXD, 2RRC, 3SKI, 430D, 3OXE, 1TOB, 4E8Q, 1VC5, 1CX0, 3OXJ, 2ZH2, 3NDB, 1FJE, 4Y1M, 1VC7, 1TN2, 4EN5, 2OE8, 3IZ4, 3OXM, 1JID, 1S2F, 2HOK, 3GX3, 1D0T, 1S34, 3P22, 1ROQ, 3PDR, 2GIS, 4ZNP, 3AL0, 2KF0, 3AMU, 2KD8, 4XNR, 1I9V, 2EES, 3AMT, 3SD3, 3Q3Z, 3RG5, 2ESI, 4FRN, 3RKF, 3NPQ, 3SKL, 1D0U, 3GX2, 2HOJ, 2XEB, 2JXV, 3G4M, 1A60, 2ZH1, 1VC6, 3SKR, 1VOP, 2ZH5, 2XDB, 1ATW, 3SKW, 3R4F, 3GX6, 3NPB, 2OIH, 1K4A, 3SLM, 3SUH, 1YFG, 3Q51, 4FE5, 2EEW, 2B57, 387D, 2EEV, 4DS6, 4E8M, 4FAR, 3D0U, 2Y95,

5BTP, 4FAU, 3GX7, 2HOO, 2OJ3, 1MNB, 1ATV, 4GXY, 2ZH4, 2ZHB, 2MFD, 1A9L, 4QJH, 1FHK, 1F1T, 2ZH6, 1ZBN, 2O3X, 2TRA, 1ZL3, 2HOM, 3GX5, 4QLM, 1K4B, 1GTR, 4WFM, 2LUP, 3OVA, 3MXH, 1KXK, 4Y1I, 4Y1J, 2L5Z, 2EET, 2NUE, 1EXD, 2RE8, 1PBR, 1P5P, 2EEU, 2LA5, 364D, 2KEZ, 2LAC, 1ETF, 3Q50, 4C4Q, 1K8W, 1GTS, 2F4T, 2HOL, 1HQ1, 2A2E, 4E8T, 2ZH7, 1VC0, 2ZHA, 2HVY, 2MF1, 4YAZX.
