# Supplementary material for: GARN3: A coarse-grained helix centered technique for RNA 3D structures prediction
Source: PLoS One. 2026 Jun 22;21(6):e0328609. doi: 10.1371/journal.pone.0328609 (PMC13286185; doi:10.1371/journal.pone.0328609)
Supplement: S2 Appendix — This section contains the evaluation previously done for the 1-bead representation in GARN, related to the scoring function update. (PDF) [file pone.0328609.s002.pdf]

S2 Appendix - Machine learning model using a GARN model with 1-bead representation

During the development of GARN3, we initially explored an alternative coarse-grained representation in which each nucleotide in the RNA molecule is represented by a single pseudoatom (1-bead per nucleotide). While the 1-bead representation offers a simpler, more uniform abstraction across all secondary structure elements, it results in a substantially larger number of players for typical RNA molecules. This increase in player count led to an exponential growth in the strategy space within the game-theoretic framework, significantly increasing computational complexity and execution time beyond practical limits for the available implementation. Therefore, before using the 1-bead representation model, the GARN modeling strategy needs to be adapted accordingly.

This appendix describes the machine learning–based scoring function developed for the 1-bead GARN model. Although this representation was not selected for the final GARN3 implementation—primarily due to the computational scalability challenges associated with the expanded strategy space—the predictive model itself demonstrated promising performance and may serve as a foundation for future work, particularly if algorithmic improvements to handle larger strategy spaces become available, or for applications such as full-atom reconstruction pipelines.

GARN 3D model with 1-bead per nucleotide representation, where the structures in cyan and white were initially used in our dataset to train and test a regression model. The coarse-grained structure in cyan and white was obtained using the experimental structure. The rose-colored structure is an example of a prediction from the GARN algorithm using a 1-bead-per-nucleotide representation. The PDB ID used for this example is 1XHP.

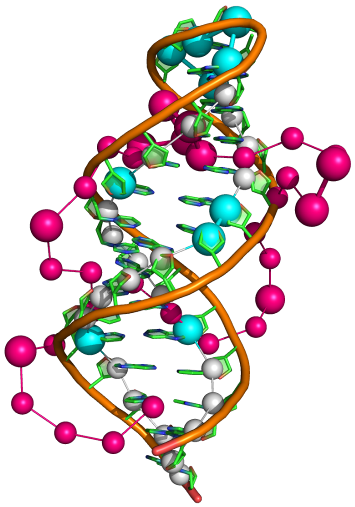

Feature Engineering for 1-Bead Representation

The scoring function for the 1-bead GARN model was developed using a machine learning regression model. Similar to the GARN3 model described in the manuscript, the objective was to predict pairwise interaction scores (distances) between pseudoatoms based on structural and topological features extracted from the secondary structures.

Because each nucleotide corresponds to exactly one pseudoatom in this

representation, the feature set required adaptation to account for the absence of distinct bead types within helices. After initial analysis, 13 features were defined to capture these relationships. The features are enumerated below:

1. SSE type of player X;
2. SSE type of player Y;
3. Shortest-path distance between players X and Y, considering that the GARN3 model is represented as a graph. For that, the Dijkstra algorithm was used;
4. If players X and Y represent nucleotides in helices and are placed at the same k-way junction branch, this feature has a value of 1; otherwise, its value is 0. If this condition is not applicable, this value is -1;
5. If players X and Y are in different stem-loops and are pseudoknots interacting (kissing loops), this value is 1; otherwise, its value is 0. If this condition is not applicable, this value is -1;
6. If players X and Y are nucleotides from the same helix and on the same side, i.e., both opening side "(" or both closing side ")" of a helix, this value is 1; otherwise, its value is 0. If this condition is not applicable, this value is -1;
7. Largest junction quantity in the molecule. For example, if the molecule has only base pairs and a stem-loop, this number is 1. If the molecule has a 2-way junction, this number is 2, and it follows the same logic for higher-order n-way junctions;
8. If players X and Y are placed in the same unpaired area, this feature has value 1, and 0 otherwise (e.g., both players are part of the same stem-loop, or part of the same 2-way junction); if not applicable, this value is -1;
9. Number of distinct k-way junctions between players X and Y;
10. If players X and Y are helices and form a base pair, this value is 1; otherwise, its value is 0. If this condition is not applicable, this value is -1;
11. Number of helices/terminal-loop separators between players X and Y;
12. If players X and Y are helices, this feature captures the number of intervening 2-way junctions between them; otherwise, this value is -1;
13. Total of players considering the entire molecule.

These features collectively encode both the local binding geometry and the global topological arrangement of the RNA structure. Features 1–3 capture basic player identity and connectivity; features 4–6 and 8 encode junction-specific and helix-specific relationships; features 9–11 represent structural motifs such as pseudoknots and base pairing; and features 7, 12, and 13 provide context regarding junction complexity and molecule size.

Model Training and Algorithm Selection

To evaluate the predictive capacity of these features, we trained multiple regression algorithms on a dataset derived from experimentally determined RNA structures. The dataset was constructed following a procedure analogous to that described in the main text: pairwise distances were extracted from native 3D coordinates mapped onto 1-bead coarse-grained representations, and the corresponding feature vectors were computed from secondary structure annotations.

The models were evaluated using 10-fold (k-fold with $k = 10$) cross-validation, ensuring that the performance metrics reflect generalization to unseen data. We assessed each algorithm using three standard regression metrics: the coefficient of determination (R^2), mean absolute error (MAE, measured in Ångströms), and root mean squared error (RMSE, also in Ångströms). Higher R^2 values indicate better explanatory power, while lower MAE and RMSE values reflect more accurate distance predictions. Table 1 summarizes the cross-validation results for ten regression algorithms.

Algorithms tested using the dataset from this study. The columns represent the R^2, MAE (Mean Absolute Error), and RMSE (Root Mean Squared Error) scores obtained after cross-validation. The values are sorted by R^2 score, in descending order.

Algorithm	R^2	MAE	RMSE
Gradient Boosting	0.86	10.14	15.23
kNN (k-Nearest Neighbors)	0.86	9.98	15.33
Random Forest	0.83	11.40	16.50
Decision Tree	0.82	11.70	17.02
Neural Networks	0.74	14.64	20.42
Linear Regression	0.65	17.59	23.82
Ridge	0.65	17.59	23.82
Lasso	0.65	17.59	23.82
Bayesian Ridge	0.65	17.59	23.82
SVM	0.58	18.86	25.96

The results from the table in this appendix show that Gradient Boosting and kNN achieve the best predictive performance, both with $R^2 = 0.86$ and MAE values of 10.14 Å and 9.98 Å, respectively, followed by Random Forest ($R^2 = 0.83$, MAE = 11.40 Å) and Decision Tree ($R^2 = 0.82$, MAE = 11.70 Å). Neural Networks achieve moderate performance ($R^2 = 0.74$, MAE = 14.64 Å), while Linear Regression, Ridge, Lasso, and Bayesian Ridge all obtain $R^2 = 0.65$, and SVM shows the poorest results ($R^2 = 0.58$, MAE = 18.86 Å), likely due to issues with hyperparameter tuning.

Despite the 1-bead model's promising predictive accuracy, the computational challenges outlined earlier—specifically the exponential growth in strategy space—made this representation impractical for GARN3. Future work could revisit this approach with algorithmic improvements to handle larger strategy spaces more efficiently, potentially enabling its use in full-atom reconstruction pipelines or hybrid coarse-graining strategies that adaptively switch between representations based on molecule size and topology.
