# Supplementary material for: GARN3: A coarse-grained helix centered technique for RNA 3D structures prediction
Source: PLoS One. 2026 Jun 22;21(6):e0328609. doi: 10.1371/journal.pone.0328609 (PMC13286185; doi:10.1371/journal.pone.0328609)

S1 Fig. Example of GARN3 graph representation. The players in blue color represent helix pseudoatoms (base-pairs). The players in yellow represent pseudoatoms in terminal loops, 2-way junctions, and 3-way junctions. Finally, the players in purple and green colors represent k-way ($k > 3$) junction pseudoatoms.

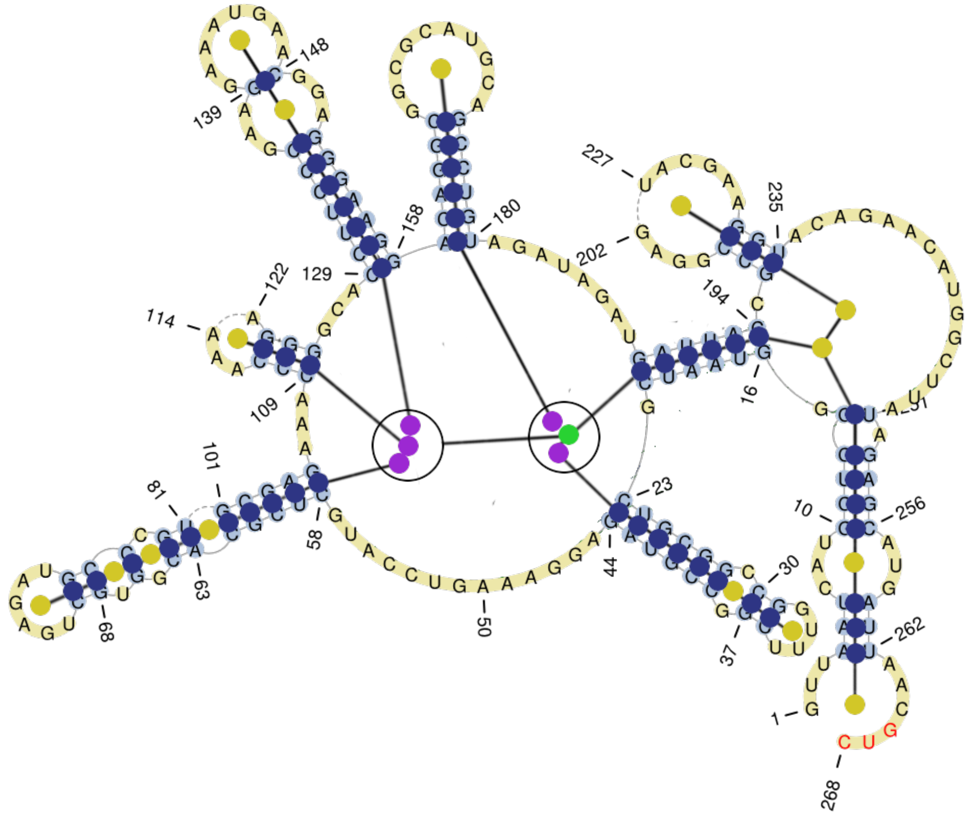

Supplement: S1 Fig — The players in blue color represent helix pseudoatoms (base-pairs). The players in yellow represent pseudoatoms in terminal loops, 2-way junctions, and 3-way junctions. Finally, the players in purple and green colors represent k-way (k > 3) junction pseudoatoms. (PDF) [file pone.0328609.s003.pdf]
