# Supplementary material for: GARN3: A coarse-grained helix centered technique for RNA 3D structures prediction
Source: PLoS One. 2026 Jun 22;21(6):e0328609. doi: 10.1371/journal.pone.0328609 (PMC13286185; doi:10.1371/journal.pone.0328609)

S3 Fig. Best simulations for the molecule 1XHP. This figure presents the best simulation for molecule 1XHP, using each of the techniques tested here. SimRNA is absent because it cannot be generated. GARN2 is also absent, as it uses a different model with fewer pseudoatoms.

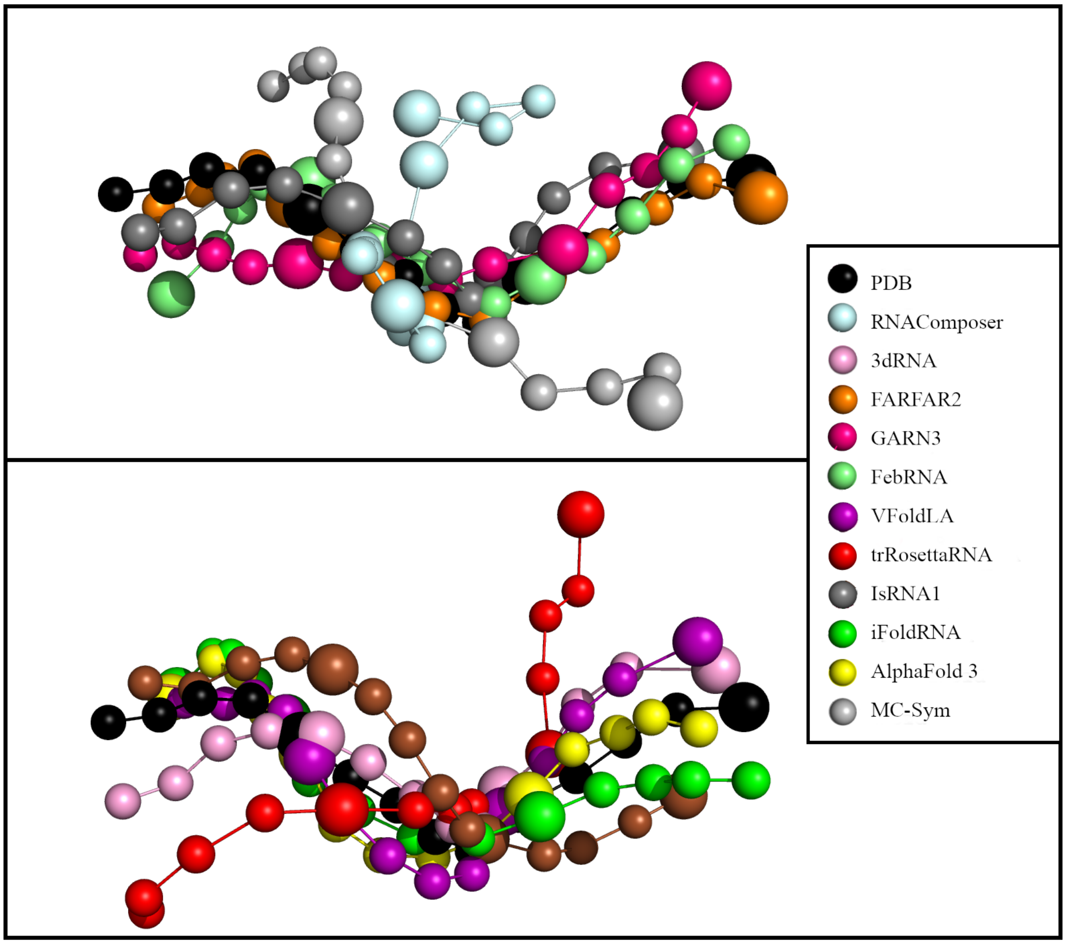

Supplement: S3 Fig — This figure presents the best simulation for molecule 1XHP, using each of the techniques tested here. SimRNA is absent because it cannot be generated. GARN2 is also absent, as it uses a different model with fewer pseudoatoms. (PDF) [file pone.0328609.s005.pdf]
