# Supplementary material for: GARN3: A coarse-grained helix centered technique for RNA 3D structures prediction
Source: PLoS One. 2026 Jun 22;21(6):e0328609. doi: 10.1371/journal.pone.0328609 (PMC13286185; doi:10.1371/journal.pone.0328609)

S5 Fig. Maximum distance of GARN3 sampling for test set B. This plot presents the highest maximum and lowest maximum distances between the nodes, for each of the molecules in the test set B.

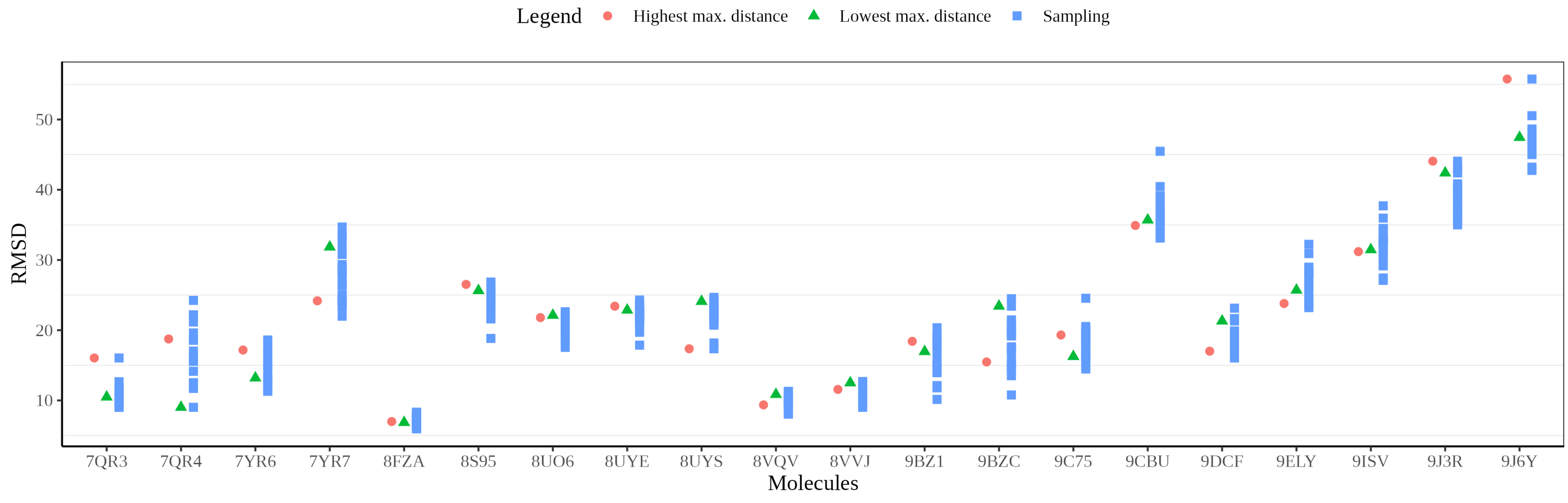

Supplement: S5 Fig — This plot presents the highest maximum and lowest maximum distances between the nodes, for each of the molecules in Test Set B. (PDF) [file pone.0328609.s007.pdf]
