# Supplementary material for: GARN3: A coarse-grained helix centered technique for RNA 3D structures prediction
Source: PLoS One. 2026 Jun 22;21(6):e0328609. doi: 10.1371/journal.pone.0328609 (PMC13286185; doi:10.1371/journal.pone.0328609)
Supplement: S1 Table — Molecules used to run the simulations and perform the evaluation. This test set contains 22 molecules, where primary and secondary structures were obtained from the RNA FRABASE repository. (PDF) [file pone.0328609.s010.pdf]

S1 Table. Test set A, using molecules from RNA FRABASE. Molecules used to run the simulations and perform the evaluation. This test set contains 22 molecules, where primary and secondary structures were obtained from the RNA FRABASE repository.

**Molecule**	**Description**	**Nucleotides**	**Players**
1XHP	Extended U6 ISL	32	14
1MNX	Loop E Region of the 5S rRNA from Spinach Chloroplasts	42	14
1CQ5	NMR structure of SRP RNA domain IV	43	14
2RP0	PEMV-1 mRNA pseudoknot	27	7
2N6S	CssA4 (bottom stem) of CssA thermometer	36	18
1Q29	Hammerhead Ribozyme with 5'-5' G-G linkage	41	16
3DIR	Crystallization of the Thermotoga maritima lysine riboswitch bound to N6-1-iminoethyl-L-Lysine	174	72
4P8Z	Speciation of a group I intron into a lariat capping ribozyme	188	67
3AM1	O-Phosphoseryl-tRNA kinase complexed with anticodon-stem/loop truncated tRNA	81	36
4RZD	Crystal Structure of a PreQ1 Riboswitch	102	31
4QKA	c-di-AMP riboswitch from Thermoanaerobacter pseudethanolicus, iridium hexamine soak	122	38
1Z43	Crystal structure of 7S.S SRP RNA of M. jannaschii	101	40
4P9R	Speciation of a group I intron into a lariat capping ribozyme	189	67
4OQU	Structure of the SAM-I/IV riboswitch	97	38
4QK8	Thermoanaerobacter pseudethanolicus c-di-AMP riboswitch	124	38
5J01	Structure of the lariat form of a chimeric derivative of the Oceanobacillus iheyensis group II intron in the presence of NH4+ and MG2+.	418	132
3J28	Dissecting the in vivo assembly of the 30S ribosomal subunit reveals the role of RimM	1533	584
1C2W	23S RRNA structure	2904	1079
2NBX	Solution structure of the J-K region of EMCV IRES	108	55
2G1W	NMR structure of the Aquifex aeolicus tmRNA pseudoknot PK1	22	6
1KAJ	RNA pseudoknot from mouse mammary tumor virus	32	6
2ZUF	Crystal structure of Pyrococcus horikoshii arginyl-tRNA synthetase complexed with tRNA	78	29
