# Supplementary material for: GARN3: A coarse-grained helix centered technique for RNA 3D structures prediction
Source: PLoS One. 2026 Jun 22;21(6):e0328609. doi: 10.1371/journal.pone.0328609 (PMC13286185; doi:10.1371/journal.pone.0328609)
Supplement: S2 Table — Molecules used to run the simulations and perform the evaluation. This test set contains 20 molecules, and it considers only RNA structures evaluated in CASP 15 and 16. Each target ID is referenced with its corresponding PDB ID and description. (PDF) [file pone.0328609.s011.pdf]

S2 Table. Test set B, using molecules from CASP (Critical Assessment of Structure Prediction) 15 and 16. Molecules used to run the simulations and perform the evaluation. This test set contains 20 molecules, and it considers only RNA structures evaluated in CASP 15 and 16. Each target ID is referenced with its corresponding PDB ID and description.

CASP	Target ID	Description	Nucleotides	Players
CASP15	R1108	CPEB3 ribozyme Chimpanzee	69	16
CASP15	R1107	CPEB3 ribozyme Human	69	16
CASP15	R1190	Pseudomonas aeruginosa RsmZ with two RsmA protein dimers	176	47
CASP15	R1189	Pseudomonas aeruginosa RsmZ with three RsmA protein dimers	176	42
CASP15	R1117	PreQ1 class I type III riboswitch	30	6
CASP15	R1116	Cloverleaf RNA	157	66
CASP15	R1203	Rev response element stem-loop II	134	55
CASP15	R1156	BtCoV-HKU5 SL5	135	63
CASP15	R1149	SARS-CoV-2 SL5	124	54
CASP15	R1263	ZTP riboswitch with m-1-pyridinyl AICA	64	23
CASP15	R1264	ZTP riboswitch with AICA derivative	64	23
CASP16	R1262v1	ZTP riboswitch with AICA	89	30
CASP16	R1261v1	ZTP riboswitch	89	30
CASP16	R1296	G34U mutant of M1209 complex	72	34
CASP16	R1260	Ribozyme solvent shell	387	144
CASP16	R1211	CVB3 cloverleaf RNA in complex with 3C protease	90	35
CASP16	R1242	RaiA RNA motif C. acetobutylicum	205	69
CASP16	R1283v1	Enterococcus ncRNA	580	72
CASP16	R1283v2	Enterococcus ncRNA	580	233
CASP16	R1286	Lactobacillus ncRNA	526	98
