# Supplementary material for: GARN3: A coarse-grained helix centered technique for RNA 3D structures prediction
Source: PLoS One. 2026 Jun 22;21(6):e0328609. doi: 10.1371/journal.pone.0328609 (PMC13286185; doi:10.1371/journal.pone.0328609)
Supplement: S3 Table — The columns represent the R2, MAE (Mean Absolute Error), and RMSE (Root Mean Squared Error) scores obtained after cross-validation. The values are sorted by R2 score, in descending order. (PDF) [file pone.0328609.s012.pdf]

S3 Table. Algorithms tested using the dataset of molecules from this study, excluding all molecules used in our predictions and evaluation. The columns represent the R^2, MAE (Mean Absolute Error), and RMSE (Root Mean Squared Error) scores obtained after cross-validation. The values are sorted by R^2 score, in descending order.

**Algorithm**	$\mathbf{R^2}$	**MAE**	**RMSE**
Gradient Boosting	0.82	6.14	9.88
Decision Tree	0.77	6.36	11.32
Random Forest	0.75	8.10	11.85
kNN (k-Nearest Neighbors)	0.75	6.65	11.77
Neural Networks	0.54	11.06	15.97
Ridge	0.31	13.87	19.50
Lasso	0.31	13.86	19.50
Linear Regression	0.31	13.87	19.50
SVM	0.12	15.12	21.96
