# Supplementary material for: GARN3: A coarse-grained helix centered technique for RNA 3D structures prediction
Source: PLoS One. 2026 Jun 22;21(6):e0328609. doi: 10.1371/journal.pone.0328609 (PMC13286185; doi:10.1371/journal.pone.0328609)
Supplement: S4 Table — Comparison of GARN3 with other techniques, considering only template-based techniques. The scores are independent, therefore the structure with best-ranked RMSD not necessarily is the one with best-ranked TM-Score. (PDF) [file pone.0328609.s013.pdf]

S4 Table. Template-based techniques simulation results from test set A. Comparison of GARN3 with other techniques, considering only template-based techniques. The scores are independent, therefore the structure with best-ranked RMSD not necessarily is the one with best-ranked TM-Score.

**Mol.**	**Type**	**Len.**	**RMSD / TM**	**FARFAR2**	**RNA-Composer**	**VFold-LA**	**3dRNA**	**FebRNA**	**MC-Sym**	**GARN3**
1XHP	2-way	32	Min	**0.92** / **0.946**	1.93 / 0.800	3.70 / 0.547	1.35 / 0.880	1.41 / 0.876	1.38 / 0.920	3.1 / 0.698
			Max	2.58 / 0.702	1.93 / 0.800	3.70 / 0.547	9.37 / 0.369	2.88 / 0.656	3.52 / 0.623	5.83 / 0.578
1MNX	2-way	42	Min	2.17 / 0.754	**1.74** / **0.869**	2.72 / 0.850	1.91 / 0.821	2.21 / 0.757	–	3.29 / 0.592
			Max	8.64 / 0.389	1.74 / 0.869	2.72 / 0.850	8.05 / 0.441	2.52 / 0.711	–	4.63 / 0.499
1CQ5	2-way	43	Min	2.62 / 0.700	8.04 / 0.355	1.01	3.35 / 0.608	**0.9** / **0.938**	–	3.25 / 0.617
			Max	6.80 / 0.330	8.04 / 0.355	1.01	13.5 / 0.293	9.45 / 0.311	–	6.26 / 0.530
2RP0	2-way	27	Min	2.25 / 0.908	4.22 / 0.902	6.68 / 0.849	**0.48** / **0.984**	1.15 / 0.911	–	6.76 / 0.774
			Max	5.81 / 0.780	4.22 / 0.902	6.68 / 0.849	11.0 / 0.850	4.35 / 0.818	–	7.68 / 0.752
2N6S	2-way	36	Min	–	2.28 / 0.751	–	**1.35** / 0.874	1.96 / 0.793	1.62 / **0.897**	3.01 / 0.627
			Max	–	2.28 / 0.751	–	2.44 / 0.775	2.13 / 0.778	4.86 / 0.536	7.22 / 0.601
1Q29	3-way	41	Min	**3.47** / **0.595**	4.91 / 0.544	–	4.42 / 0.534	–	–	9 / 0.354
			Max	7.83 / 0.388	4.91 / 0.544	–	8.85 / 0.370	–	–	11 / 0.421
3DIR	3-way	174	Min	12.7 / 0.388	**0.6** / **0.988**	19.7 / 0.451	17.8 / 0.354	6.31 / 0.624	–	14.5 / 0.304
			Max	24.8 / 0.230	0.6 / 0.988	19.7 / 0.451	31.7 / 0.161	16.9 / 0.323	–	24.1 / 0.268
4P8Z	3-way	188	Min	15.7 / 0.343	26.7 / 0.210	–	–	**6.41** / **0.486**	–	18.6 / 0.380
			Max	29.1 / 0.251	26.7 / 0.210	–	–	9.02 / 0.450	–	27.5 / 0.234
3AM1	3-way	81	Min	10.7 / 0.510	1.23 / 0.913	15.5 / 0.349	**0.73** / **0.975**	–	-	12.9 / 0.350
			Max	18.8 / 0.363	1.23 / 0.913	15.5 / 0.349	1.48 / 0.952	–	-	16.7 / 0.342
4RZD	3-way	102	Min	**7.35** / **0.449**	12.5 / 0.411	14.2 / 0.358	21.1 / 0.383	–	–	10.1 / 0.376
			Max	9.98 / 0.341	12.5 / 0.411	14.2 / 0.358	64.2 / 0.202	–	–	16.4 / 0.377
4QKA	3-way	122	Min	12.3 / 0.406	14.4 / 0.330	12.3 / 0.398	17.0 / 0.342	–	–	11.2 / 0.382
			Max	17.3 / 0.309	14.4 / 0.330	12.3 / 0.398	48.2 / 0.220	–	–	19.9 / 0.357
1Z43	3-way	101	Min	8.23 / 0.570	**2.6** / **0.756**	28.9 / 0.325	11.9 / 0.430	–	–	11.8 / 0.348
			Max	29.6 / 0.311	2.6 / 0.756	28.9 / 0.325	30.4 / 0.292	–	–	22 / 0.319
4P9R	3-way	189	Min	27.6 / **0.403**	26.6 / 0.234	–	28.2 / 0.367	–	–	19.9 / 0.297
			Max	32.4 / 0.245	26.6 / 0.234	–	91.6 / 0.043	–	–	33.9 / 0.237
4OQU	n-way	97	Min	9.71 / 0.444	18.4 / 0.378	–	**5.65** / **0.720**	–	–	11.6 / 0.348
			Max	18.9 / 0.319	18.4 / 0.378	–	23.8 / 0.285	–	–	18.2 / 0.277
4QK8	n-way	124	Min	**10.8** / 0.398	14.5 / 0.330	12.4 / 0.395	17.0 / 0.340	–	–	9.43 / 0.344
			Max	17.3 / 0.322	14.5 / 0.330	12.4 / 0.395	48.2 / 0.220	–	–	19.9 / 0.355
5J01	n-way	418	Min	38.6 / 0.164	42.8 / **0.255**	–	39.5 / 0.199	–	–	30.1 / 0.165
			Max	56.9 / 0.098	42.8 / 0.255	–	87.6 / 0.059	–	–	36.8 / 0.157
3J28	n-way	1533	Min	–	–	–	–	–	–	53.2 / 0.054
			Max	–	–	–	–	–	–	68 / 0.056
1C2W	n-way	2904	Min	–	–	–	–	–	–	67.1 / 0.038
			Max	–	–	–	–	–	–	82.4 / 0.025
2NBX	n-way	108	Min	–	11.9 / 0.405	11.1 / 0.350	**2.91** / **0.832**	20.1 / 0.423	8.75 / 0.487	16 / 0.303
			Max	–	11.9 / 0.405	11.1 / 0.350	21.3 / 0.320	24.7 / 0.334	21.1 / 0.311	20.8 / 0.369
2G1W	pseudo-knot	22	Min	1.60 / 0.893	2.56 / 0.833	2.15 / 0.864	**1.21** / **0.916**	–	–	5.46 / 0.834
			Max	3.38 / 0.729	2.56 / 0.833	2.15 / 0.864	14.7 / 0.785	–	–	7.59 / 0.810
1KAJ	pseudo-knot	32	Min	1.44 / 0.925	4.74 / 0.861	**1.01** / 0.933	1.24 / **0.934**	–	–	6.77 / 0.776
			Max	4.98 / 0.809	4.74 / 0.861	1.01 / 0.933	5.00 / 0.827	–	–	10.7 / 0.779
2ZUF	pseudo-knot	78	Min	11.6 / 0.406	15.7 / 0.394	16.8 / 0.397	15.6 / 0.399	–	–	6.6 / 0.359
			Max	18.4 / 0.339	15.7 / 0.394	16.8 / 0.397	18.0 / 0.365	–	–	8.88 / 0.391
