# Supplementary material for: GARN3: A coarse-grained helix centered technique for RNA 3D structures prediction
Source: PLoS One. 2026 Jun 22;21(6):e0328609. doi: 10.1371/journal.pone.0328609 (PMC13286185; doi:10.1371/journal.pone.0328609)
Supplement: S5 Table — Comparison of GARN3 with other techniques, considering only template-based techniques. The scores are independent, therefore the structure with best-ranked RMSD not necessarily is the one with best-ranked TM-Score. (PDF) [file pone.0328609.s014.pdf]

S5 Table. Template-based techniques simulation results from test set B. Comparison of GARN3 with other techniques, considering only template-based techniques. The scores are independent, therefore the structure with best-ranked RMSD not necessarily is the one with best-ranked TM-Score.

Mol.	Type	Len.	RMSD / TM	FARFAR2	RNA-Composer	Vfold	3dRNA	FebRNA	MC-Sym	GARN3
8VQV	2-way	64	Min	4.59 / 0.621	-	-	4.73 / 0.654	**3.0** / **0.724**	-	8.05 / 0.418
			Max	12.94 / 0.38	-	-	10.57 / **0.443**	**9.12** / 0.424	-	11.3 / 0.362
8VVJ	2-way	64	Min	4.91 / **0.675**	10.31 / 0.443	-	**4.84** / 0.667	-	-	9.02 / 0.493
			Max	13.27 / 0.387	13.07 / 0.343	-	**10.61** / **0.446**	-	-	12.7 / 0.458
9BZ1	2-way	89	Min	**5.15** / **0.61**	6.57 / 0.567	-	18.0 / 0.436	-	-	10.1 / 0.426
			Max	14.79 / 0.341	**9.83** / **0.419**	-	25.62 / 0.31	-	-	20.4 / 0.359
9BZC	2-way	89	Min	**3.75** / **0.678**	6.47 / 0.538	-	18.25 / 0.438	-	-	10.8 / 0.331
			Max	15.95 / 0.319	**11.49** / **0.38**	-	25.3 / 0.326	-	-	24.5 / 0.377
7YR6	2-way	176	Min	19.39 / 0.347	24.15 / 0.288	-	28.29 / 0.313	-	-	11.3 / 0.358
			Max	28.04 / 0.202	37.41 / 0.188	-	107.11 / 0.115	-	-	18.6 / 0.339
7YR7	2-way	176	Min	19.8 / 0.327	20.92 / 0.32	-	19.3 / 0.32	-	-	22 / 0.252
			Max	**30.48** / 0.168	33.28 / **0.206**	-	44.02 / 0.157	-	-	34.7 / 0.389
9C75	3-way	72	Min	17.52 / 0.355	-	-	**14.18** / 0.342	-	17.38 / 0.335	14.5 / 0.355
			Max	19.47 / 0.225	-	-	**17.7** / **0.327**	-	21.39 / 0.247	24.5 / 0.399
9ELY	3-way	205	Min	29.06 / **0.354**	-	-	25.21 / 0.285	-	-	23.2 / 0.280
			Max	54.88 / 0.15	-	-	**36.13** / **0.159**	-	-	32.2 / 0.214
9DCF	n-way	90	Min	-	-	-	14.68 / 0.391	-	19.85 / 0.39	16 / 0.391
			Max	-	-	-	22.15 / 0.304	-	20.21 / **0.364**	23.1 / 0.220
8UYS	n-way	124	Min	**10.45** / **0.477**	15.99 / 0.378	-	16.06 / 0.424	-	-	17.4 / 0.333
			Max	20.4 / 0.29	**17.32** / **0.322**	-	25.9 / 0.297	-	-	24.7 / 0.269
8UO6	n-way	134	Min	14.79 / 0.401	16.64 / 0.496	-	**10.18** / **0.57**	-	-	17.6 / 0.367
			Max	26.41 / 0.187	**17.94** / 0.424	-	26.09 / **0.442**	-	-	22.6 / 0.341
8UYE	n-way	135	Min	**8.11** / **0.539**	24.5 / 0.367	-	19.52 / 0.367	17.29 / 0.399	-	17.9 / 0.257
			Max	29.13 / 0.283	27.7 / 0.298	-	32.38 / 0.22	**20.31** / **0.337**	-	24.3 / 0.222
8S95	n-way	157	Min	-	-	-	**11.25** / **0.468**	-	-	18.8 / 0.295
			Max	-	-	-	**21.66** / **0.367**	-	-	26.9 / 0.326
9CBU	n-way	387	Min	37.93 / **0.287**	-	-	39.35 / 0.157	-	-	33.1 / 0.186
			Max	59.63 / **0.127**	-	-	104.97 / 0.041	-	-	45.5 / 0.135
9J6Y	n-way	526	Min	-	-	-	-	-	-	42.8 / 0.152
			Max	-	-	-	-	-	-	55.8 / 0.106
9ISV	n-way	580	Min	-	-	-	-	-	-	27.1 / 0.292
			Max	-	-	-	-	-	-	37.7 / 0.250
9J3R	n-way	580	Min	-	-	-	-	-	-	35 / 0.146
			Max	-	-	-	-	-	-	44.1 / 0.108
8FZA	p-knot	30	Min	-	9.94 / **0.819**	-	**3.25** / 0.718	-	-	5.95 / 0.742
			Max	-	11.4 / 0.62	-	**7.3** / 0.654	-	-	8.33 / 0.813
7QR3	p-knot	69	Min	**11.44** / 0.457	18.72 / 0.331	-	13.16 / **0.46**	-	-	9.01 / 0.364
			Max	**16.23** / 0.329	18.72 / 0.331	-	36.83 / **0.364**	-	-	16 / 0.434
7QR4	p-knot	69	Min	12.51 / **0.452**	17.1 / 0.39	-	**12.18** / 0.443	-	-	9.03 / 0.418
			Max	**15.39** / 0.313	18.53 / 0.329	-	25.98 / 0.34	-	-	24.2 / 0.408
