# Supplementary material for: GARN3: A coarse-grained helix centered technique for RNA 3D structures prediction
Source: PLoS One. 2026 Jun 22;21(6):e0328609. doi: 10.1371/journal.pone.0328609 (PMC13286185; doi:10.1371/journal.pone.0328609)
Supplement: S6 Table — Comparison of GARN3 with other techniques, considering only techniques based on a deep learning approach. The scores are independent, therefore the structure with best-ranked RMSD not necessarily is the one with best-ranked TM-Score. (PDF) [file pone.0328609.s015.pdf]

S6 Table. Deep learning-based techniques' simulation results from test set A. Comparison of GARN3 with other techniques, considering only techniques based on the deep learning approach. The scores are independent, therefore the structure with best-ranked RMSD not necessarily is the one with best-ranked TM-Score.

Mol.	Type	Len.	RMSD / TM	AlphaFold	trRosettaRNA	GARN3
1XHP	2-way	32	Min	**1.82** / **0.823**	2.75 / 0.719	3.1 / 0.698
			Max	2.36 / 0.643	4.72 / 0.573	5.83 / 0.578
1MNX	2-way	42	Min	3.79 / 0.735	**2.25** / **0.800**	3.29 / 0.592
			Max	4.81 / 0.458	6.09 / 0.416	4.63 / 0.499
1CQ5	2-way	43	Min	3.12 / **0.836**	3.14 / 0.679	3.25 / 0.617
			Max	11.6 / 0.376	4.94 / 0.577	6.26 / 0.530
2RP0	2-way	27	Min	1.76 / 0.905	**0.79** / **0.955**	6.76 / 0.774
			Max	1.87 / 0.884	1.13 / 0.923	7.68 / 0.752
2N6S	2-way	36	Min	2.37 / 0.735	**0.86** / **0.950**	3.01 / 0.627
			Max	2.80 / 0.610	6.43 / 0.546	7.22 / 0.601
1Q29	3-way	41	Min	9.49 / 0.506	**3.66** / **0.559**	9 / 0.354
			Max	9.77 / 0.461	5.01 / 0.461	11 / 0.421
3DIR	3-way	174	Min	2.07 / **0.891**	**1.99** / 0.888	14.5 / 0.304
			Max	2.77 / 0.747	16.5 / 0.414	24.1 / 0.268
4P8Z	3-way	188	Min	7.50 / 0.567	**3.85** / **0.682**	18.6 / 0.380
			Max	22.1 / 0.269	26.1 / 0.374	27.5 / 0.234
3AM1	3-way	81	Min	1.74 / 0.849	**1.11** / **0.920**	12.9 / 0.350
			Max	2.19 / 0.706	11.8 / 0.515	16.7 / 0.342
4RZD	3-way	102	Min	8.66 / 0.484	**3.40** / **0.663**	10.1 / 0.376
			Max	9.37 / 0.326	9.89 / 0.363	16.4 / 0.377
4QKA	3-way	122	Min	**1.26** / **0.928**	2.48 / 0.700	11.2 / 0.382
			Max	1.85 / 0.858	14.0 / 0.363	19.9 / 0.357
1Z43	3-way	101	Min	1.30 / 0.914	**1.03** / **0.934**	11.8 / 0.348
			Max	1.82 / 0.806	1.3 / 0.903	22 / 0.319
4P9R	3-way	189	Min	26.6 / 0.493	25.9 / **0.508**	19.9 / 0.297
			Max	39.9 / 0.209	29.8 / 0.316	33.9 / 0.237
4OQU	n-way	97	Min	13.2 / 0.410	**1.47** / **0.878**	11.6 / 0.348
			Max	14.3 / 0.314	15.8 / 0.376	18.2 / 0.277
4QK8	n-way	124	Min	1.30 / **0.934**	**1.25** / 0.905	9.43 / 0.344
			Max	1.56 / 0.857	13.8 / 0.409	19.9 / 0.355
5J01	n-way	418	Min	–	–	30.1 / 0.165
			Max	–	–	36.8 / 0.157
3J28	n-way	1533	Min	**5.46** / **0.777**	–	53.2 / 0.054
			Max	6.28 / 0.722	–	68 / 0.056
1C2W	n-way	2904	Min	**0.8** / **0.991**	–	67.1 / 0.038
			Max	11.8 / 0.98	–	82.4 / 0.025
2NBX	n-way	108	Min	8.36 / **0.503**	**6.18** / 0.484	16 / 0.303
			Max	9.94 / 0.347	9.0 / 0.392	20.8 / 0.369
2G1W	pseudo-knot	22	Min	2.57 / 0.842	**2.21** / **0.871**	5.46 / 0.834
			Max	2.74 / 0.708	2.37 / 0.749	7.59 / 0.810
1KAJ	pseudo-knot	32	Min	1.38 / 0.907	**0.6** / **0.976**	6.77 / 0.776
			Max	3.27 / 0.627	1.59 / 0.922	10.7 / 0.779
2ZUF	pseudo-knot	78	Min	15.7 / 0.412	15.6 / 0.400	6.6 / 0.359
			Max	15.8 / 0.404	15.9 / 0.334	8.88 / 0.391
