# Supplementary material for: GARN3: A coarse-grained helix centered technique for RNA 3D structures prediction
Source: PLoS One. 2026 Jun 22;21(6):e0328609. doi: 10.1371/journal.pone.0328609 (PMC13286185; doi:10.1371/journal.pone.0328609)
Supplement: S7 Table — Comparison of GARN3 with other techniques, considering only techniques based on a deep learning approach. The scores are independent, therefore the structure with best-ranked RMSD not necessarily is the one with best-ranked TM-Score. (PDF) [file pone.0328609.s016.pdf]

S7 Table. Simulation results for deep learning-based techniques on test set B. Comparison of GARN3 with other techniques, considering only techniques based on a deep learning approach. The scores are independent, therefore the structure with best-ranked RMSD not necessarily is the one with best-ranked TM-Score.

**Mol.**	**Type**	**Len.**	**RMSD/ TM**	**AlphaFold**	**trRosettaRNA**	**GARN3**
8VQV	2-way	64	Min	1.88 / 0.858	**0.59** / **0.978**	8.05 / 0.418
			Max	11.64 / 0.598	**0.61** / **0.976**	11.3 / 0.362
8VVJ	2-way	64	Min	1.82 / 0.892	**0.91** / **0.966**	9.02 / 0.493
			Max	13.42 / 0.636	**0.92** / **0.964**	12.7 / 0.458
9BZ1	2-way	89	Min	7.57 / 0.578	**1.82** / **0.958**	10.1 / 0.426
			Max	12.88 / 0.444	**1.84** / **0.954**	20.4 / 0.359
9BZC	2-way	89	Min	5.88 / 0.6	**1.0** / **0.937**	10.8 / 0.331
			Max	8.46 / 0.522	**1.02** / **0.935**	24.5 / 0.377
7YR6	2-way	176	Min	16.7 / 0.336	26.34 / 0.197	11.3 / 0.358
			Max	44.44 / 0.144	26.37 / 0.196	18.6 / 0.339
7YR7	2-way	176	Min	**16.15** / **0.42**	20.4 / 0.357	22 / 0.252
			Max	27.9 / 0.249	**20.41** / **0.357**	34.7 / 0.389
9C75	3-way	72	Min	16.63 / 0.301	16.36 / 0.239	14.5 / 0.355
			Max	17.12 / **0.281**	**16.41** / 0.238	24.5 / 0.399
9ELY	3-way	205	Min	22.82 / 0.313	**16.36** / **0.386**	23.2 / 0.280
			Max	**23.47** / 0.298	30.03 / **0.306**	32.2 / 0.214
9DCF	n-way	90	Min	18.4 / 0.349	15.71 / 0.338	16 / 0.391
			Max	18.91 / 0.334	**15.73** / **0.337**	23.1 / 0.220
8UYS	n-way	124	Min	20.52 / 0.425	**2.11** / **0.862**	17.4 / 0.333
			Max	25.2 / 0.275	**2.13** / **0.86**	24.7 / 0.269
8UO6	n-way	134	Min	**8.73** / **0.568**	25.36 / 0.522	17.6 / 0.367
			Max	27.76 / 0.433	**25.4** / **0.52**	22.6 / 0.341
8UYE	n-way	135	Min	25.66 / 0.452	**2.72** / **0.827**	17.9 / 0.257
			Max	27.42 / 0.424	**2.73** / **0.827**	24.3 / 0.222
8S95	n-way	157	Min	13.37 / 0.432	**5.44** / **0.601**	18.8 / 0.295
			Max	28.07 / 0.32	**5.46** / **0.6**	26.9 / 0.326
9CBU	n-way	387	Min	-	**1.69** / **0.95**	33.1 / 0.186
			Max	-	**1.71** / **0.949**	45.5 / 0.135
9J6Y	n-way	526	Min	-	36.87 / 0.151	42.8 / 0.152
			Max	-	90.48 / 0.075	55.8 / 0.106
9ISV	n-way	580	Min	-	-	27.1 / 0.292
			Max	-	-	37.7 / 0.250
9J3R	n-way	580	Min	-	-	35 / 0.146
			Max	-	-	44.1 / 0.108
8FZA	p-knot	30	Min	1.04 / 0.952	**0.81** / **0.962**	5.95 / 0.742
			Max	**1.32** / **0.936**	2.38 / 0.778	8.33 / 0.813
7QR3	p-knot	69	Min	**10.22** / **0.448**	13.83 / 0.431	9.01 / 0.364
			Max	**12.85** / 0.36	13.85 / **0.43**	16 / 0.434
7QR4	p-knot	69	Min	**12.58** / 0.425	13.86 / 0.44	9.03 / 0.418
			Max	**13.7** / 0.41	13.92 / **0.438**	24.2 / 0.408
