# Supplementary material for: GARN3: A coarse-grained helix centered technique for RNA 3D structures prediction
Source: PLoS One. 2026 Jun 22;21(6):e0328609. doi: 10.1371/journal.pone.0328609 (PMC13286185; doi:10.1371/journal.pone.0328609)
Supplement: S8 Table — Comparison of GARN3 simulations using the UCB and EXP3 algorithms. (PDF) [file pone.0328609.s017.pdf]

S8 Table. Simulations using both regret minimization algorithms in test set A. Comparison of GARN3 simulations using UCB and EXP3 algorithms.

Molecule	Type	Length	Players	RMSD	EXP3	UCB
1XHP	2-way	32	14	Min	3.08	2.91
				Max	6.63	7.59
1MNX	2-way	42	14	Min	3.39	3.23
				Max	5.4	5.29
1CQ5	2-way	43	14	Min	3.18	3.33
				Max	11.83	12.46
2RP0	2-way	27	7	Min	5.67	5.76
				Max	6.99	6.86
2N6S	2-way	36	18	Min	3.55	2.92
				Max	7.82	10.47
1Q29	3-way	41	16	Min	8.52	8.8
				Max	10.82	10.81
3DIR	3-way	174	72	Min	20.01	14.24
				Max	32.36	26.68
4P8Z	3-way	188	67	Min	17.91	19.47
				Max	27.69	31.76
3AM1	3-way	81	36	Min	12.25	12.32
				Max	18.83	18.26
4RZD	3-way	102	31	Min	11.5	9.83
				Max	19.56	20.27
4QKA	3-way	122	38	Min	13.37	10.97
				Max	22.28	23.43
1Z43	3-way	101	40	Min	11.46	14.27
				Max	24.15	31.23
4P9R	3-way	189	67	Min	19.05	19.23
				Max	32.26	31.32
4OQU	n-way	97	38	Min	12.05	10.45
				Max	20.02	20.27
4QK8	n-way	124	38	Min	11.72	9.35
				Max	26.35	22.45
5J01	n-way	418	132	Min	29.22	30.95
				Max	36.68	36.3
3J28	n-way	1533	527	Min	52.25	–
				Max	68.11	–
1C2W	n-way	2904	1079	Min	66.46	–
				Max	80.92	–
2NBX	n-way	108	55	Min	15.44	15.46
				Max	24.41	23.66
2G1W	pseudoknot	22	6	Min	4.28	4.07
				Max	6.22	6.31
1KAJ	pseudoknot	32	6	Min	5.14	4.8
				Max	8.86	8.76
2ZUF	pseudoknot	78	29	Min	6.51	6.16
				Max	11.79	12.13
