# Supplementary material for: GARN3: A coarse-grained helix centered technique for RNA 3D structures prediction
Source: PLoS One. 2026 Jun 22;21(6):e0328609. doi: 10.1371/journal.pone.0328609 (PMC13286185; doi:10.1371/journal.pone.0328609)
Supplement: S9 Table — Comparison of GARN3 simulations using the UCB and EXP3 algorithms. (PDF) [file pone.0328609.s018.pdf]

S9 Table. Simulations using both regret minimization algorithms in test set B. Comparison of GARN3 simulations using UCB and EXP3 algorithms.

Molecule	Type	Length	Players	RMSD	EXP3	UCB
7QR3	pseudoknot	69	16	Min	8.57	8.31
				Max	16.59	17.03
7QR4	pseudoknot	69	16	Min	8.27	11.52
				Max	21.99	22.84
7YR6	2-way	176	47	Min	10.97	–
				Max	17.61	–
7YR7	2-way	176	42	Min	20.75	–
				Max	28.75	–
8FZA	pseudoknot	30	6	Min	4.95	4.73
				Max	6.57	6.9
8S95	n-way	157	66	Min	18.55	21.34
				Max	33.89	35.37
8UO6	n-way	134	55	Min	18.33	16.52
				Max	27.72	27.31
8UYE	n-way	135	63	Min	19.85	17.73
				Max	28.62	24.99
8UYS	n-way	124	54	Min	17.79	16.98
				Max	28.78	28.93
8VQV	2-way	64	23	Min	7.91	7.87
				Max	17.32	17.72
8VVJ	2-way	64	23	Min	8.93	8.58
				Max	18.33	20.0
9BZ1	2-way	89	30	Min	11.31	9.16
				Max	22.42	22.9
9BZC	2-way	89	30	Min	10.41	12.97
				Max	23.94	21.57
9C75	3-way	72	34	Min	14.68	14.04
				Max	21.72	21.84
9CBU	n-way	387	144	Min	31.76	28.87
				Max	42.58	42.38
9DCF	n-way	90	35	Min	15.7	16.15
				Max	21.8	20.75
9ELY	3-way	205	69	Min	22.05	22.74
				Max	32.17	34.49
9ISV	n-way	580	72	Min	24.86	27.43
				Max	35.7	41.23
9J3R	n-way	580	233	Min	34.75	36.85
				Max	45.85	42.03
9J6Y	n-way	526	98	Min	42.75	41.07
				Max	58.11	53.12
