# Supplementary material for: GARN3: A coarse-grained helix centered technique for RNA 3D structures prediction
Source: PLoS One. 2026 Jun 22;21(6):e0328609. doi: 10.1371/journal.pone.0328609 (PMC13286185; doi:10.1371/journal.pone.0328609)
Supplement: S10 Table — The asterisk (*) next to the technique name indicates that the simulations were run on a local machine, while the others were run on its dedicated web server. The VFoldLA is not present because, even when providing different email addresses from different domains, the web server did not send any notification when the simulations finished. (PDF) [file pone.0328609.s019.pdf]

S10 Table. Simulation time of the molecules from test set A. The asterisk (*) next to the technique name indicates that the simulations were run on a local machine, while the others were run on its dedicated web server. The VFoldLA is not present because, even when providing different email addresses from different domains, the web server did not send any notification when the simulations finished.

Molecule	#	FAR-FAR2	iFold-RNA	NAST*	RNA-Composer	SimRNA	3dRNA	isRNA1	Feb-RNA*	Alpha-Fold	MC--Sym	trRo-setta-RNA	GARN 2*	GARN 3*
1XHP	32	~16m	~13m	~1m	~1m	NA	~47m	~2h	~1m	~3m	~25m	~10m	~1m	~1m
1MNX	42	~19m	~18m	~1m	~1m	~1h25	~52m	~4h	~1m	~5m	NA	~10m	~1m	~1m
1CQ5	43	~20m	~27m	~1m	~1m	NA	~52m	~4h	~1m	~5m	NA	~1m	~1m	~1m
2RP0	27	~22m	~13m	~1m	~1m	~1h	~1h22	~2h	~1m	~3m	NA	~1m	~1m	~1m
2N6S	36	NA	NA	~1m	~1m	~1h20	~44m	~1h	~1m	~3m	~30m	~1m	NA	~1m
1Q29	41	~34m	~19m	~1m	~1m	~10m	~2h	NA	~1m	~5m	NA	~10m	~1m	~1m
3DIR	174	~3h13	~2h08	~1m	~1m	~12h26	~10h12	~1d	~4m	~10m	NA	~8m	~1m	~14m
4P8Z	188	~6d10h	~2h13	~1m	~9m	~13h05	~2h18	NA	~2m	~11m	NA	~1h	NA	~12m
3AM1	81	~35m	~1h30	~1m	~3m	~1d7h	~1h07	~6h30	~1m	~9m	~32m	~46m	~1m	~15m
4RZD	102	~46m	~48m	~1m	~3m	~4h49	~6h45	~13h20	~1m	~8m	NA	~26m	~1m	~1m
4QKA	122	~1h58	~1h52	~1m	~5m	~6h	~6h20	~12h	~1m	~6m	NA	~54m	~1m	~2m
1Z43	101	~52m	~1h	~1m	~4m	NA	~1h42	~8h16	~1m	~10m	NA	~42m	~1m	~3m
4P9R	189	~6h59	~2h15	~1m	~9m	NA	~11h22	NA	~2m	~10m	NA	~1h24	~1m	~13m
4OQU	97	~1h42	~50m	~1m	~3m	~3h12	~4h39	NA	~1m	~9m	NA	~54m	~1m	~2m
4QK8	124	~10h29	~1h15	~1m	~5m	NA	~6h41	NA	~1m	~7m	NA	~58m	~1m	~2m
5J01	418	~12h34	~5h33	~10m	~24m	NA	~1d20h	NA	NA	NA	NA	~2h46	~1m	~9m
3J28	1533	NA	~1d9h	~14h05	NA	NA	NA	NA	NA	NA	NA	NA	~1m	~2h51
1C2W	2904	NA	NA	NA	NA	NA	NA	NA	NA	NA	NA	NA	~1m	~3h18
2NBX	108	NA	NA	~1m	~4m	~1d6h	~2h04	~9h44	~2m	~15m	~33m	~38m	~1m	~9m
2G1W	22	~15m	~8m	~1m	~1m	~35m	~1h24	~3h13	~1m	~2m	NA	~21m	~1m	~1m
1KAJ	32	~20m	~12m	~1m	~1m	~32m	~2h05	~3h48	~1m	~3m	NA	~24m	~1m	~1m
2ZUF	78	~7h30	~36m	~1m	~2m	~2h40	~1h14	~7h34	~1m	~7m	NA	~1h33	~1m	~1m
