# Supplementary material for: GARN3: A coarse-grained helix centered technique for RNA 3D structures prediction
Source: PLoS One. 2026 Jun 22;21(6):e0328609. doi: 10.1371/journal.pone.0328609 (PMC13286185; doi:10.1371/journal.pone.0328609)
Supplement: S12 Table — Quantity of players used when simulating the molecules in Test Set A, considering GARN2 and GARN3 models. (PDF) [file pone.0328609.s021.pdf]

S12 Table. Players in GARN2 and GARN3 models for test set A. Quantity of players used when simulating the molecules in the test set A, considering GARN2 and GARN3 models.

**Molecule**	**Nucleotides**	**Players GARN2**	**Players GARN3**
1XHP	32	6	14
1MNX	42	5	14
1CQ5	43	6	14
2RP0	27	3	7
2N6S	36	4	18
1Q29	41	8	16
3DIR	174	32	72
4P8Z	188	NA	67
3AM1	81	15	36
4RZD	102	13	31
4QKA	122	20	38
1Z43	101	18	40
4P9R	189	30	67
4OQU	97	18	38
4QK8	124	20	38
5J01	418	66	132
3J28	1533	310	584
1C2W	2904	505	1079
2NBX	108	24	55
2G1W	22	3	6
1KAJ	32	3	6
2ZUF	78	13	29
