# Supplementary material for: GARN3: A coarse-grained helix centered technique for RNA 3D structures prediction
Source: PLoS One. 2026 Jun 22;21(6):e0328609. doi: 10.1371/journal.pone.0328609 (PMC13286185; doi:10.1371/journal.pone.0328609)
Supplement: S13 Table — Quantity of players used when simulating the molecules in Test Set B, considering GARN2 and GARN3 models. The empty values in GARN2 indicate that the technique returned errors when the molecules were used as input. (PDF) [file pone.0328609.s022.pdf]

S13 Table. Players in GARN2 and GARN3 models for test set B. Quantity of players used when simulating the molecules in the test set B, considering GARN2 and GARN3 models. The empty values in GARN2 indicate that the technique returned errors when the molecules were used as input.

**Molecule**	**Nucleotides**	**Players GARN2**	**Players GARN3**
8FZA	30	3	6
8VQV	64	–	23
8VVJ	64	–	23
7QR4	69	7	16
7QR3	69	7	16
9C75	72	14	34
9BZC	89	13	30
9BZ1	89	13	30
9DCF	90	16	35
8UYS	124	24	54
8UO6	134	23	55
8UYE	135	29	63
8S95	157	29	66
7YR7	176	22	42
7YR6	176	24	47
9ELY	205	41	69
9CBU	387	70	144
9J6Y	526	47	188
9ISV	580	–	72
9J3R	580	35	233
