# Supplementary material for: GARN3: A coarse-grained helix centered technique for RNA 3D structures prediction
Source: PLoS One. 2026 Jun 22;21(6):e0328609. doi: 10.1371/journal.pone.0328609 (PMC13286185; doi:10.1371/journal.pone.0328609)

S2 Fig. GARN players ordered. The players of the highest-order junction (in the example, it is a 3-way junction) play first, and the order of the other players is defined by applying a depth-first search algorithm, starting the search from the first highest-order junction.

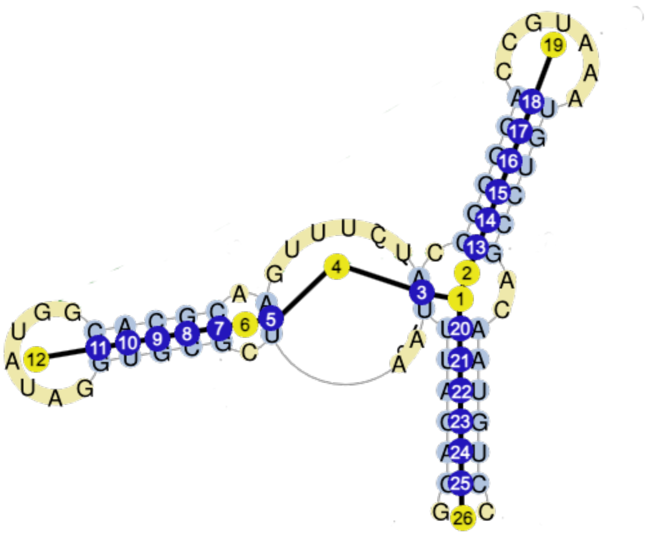

Supplement: S2 Fig — The players of the highest-order junction (in the example, it is a 3-way junction) play first, and the order of the other players is defined by applying a depth-first search algorithm, starting the search from the first highest-order junction. (PDF) [file pone.0328609.s004.pdf]
