# Supplementary material for: GARN3: A coarse-grained helix centered technique for RNA 3D structures prediction
Source: PLoS One. 2026 Jun 22;21(6):e0328609. doi: 10.1371/journal.pone.0328609 (PMC13286185; doi:10.1371/journal.pone.0328609)
Supplement: S11 Table — The asterisk (*) next to the technique name indicates that the simulations were run on a local machine, while the others were run on its dedicated web server. The VFoldLA is not present because, even when providing different email addresses from different domains, the web server did not send any notification when the simulations finished. (PDF) [file pone.0328609.s020.pdf]

S11 Table. Simulation time of the molecules from test set B. The asterisk (*) next to the technique name indicates that the simulations were run on a local machine, while the others were run on its dedicated web server. The VFoldLA is not present because, even when providing different email addresses from different domains, the web server did not send any notification when the simulations finished.

**Molecule**	**RNA Composer***	**GARN3**	**Alpha Fold***	**3d RNA***	**trRosetta RNA***	**MC Sym***	**FAR FAR2***	**iFold RNA***	**FebRNA**	**GARN2**	**NAST**	**Vfold LA***
7QR3	~3m	~2s	NA	~25m	~47m	NA	~3h	~36m	NA	~2s	~7s	NA
7QR4	~3m	~2s	NA	~25m	~47m	NA	~2h	~34m	NA	~1s	~8s	NA
7YR6	~6m	~13s	~13m	~1h	~1h	NA	~3h	~53m	NA	~26s	~5s	NA
7YR7	~6m	~17s	~6m	~1h	~58m	NA	~1h	~54m	NA	~18s	~28s	NA
8FZA	~1m	~1s	~3m	~5m	~32m	NA	NA	NA	~33s	~0s	~1s	NA
8S95	NA	~5s	~19m	~44m	~36m	NA	NA	NA	NA	~4s	~44s	NA
8UO6	~7m	~22s	~8m	~59m	~47m	NA	~10h	~1h	NA	~23s	~1m	NA
8UYE	~6m	~31s	~13m	~47m	~38m	NA	~2h	~1h	~5m	~17s	~28s	NA
8UYS	~5m	~20s	~16m	~33m	~1h	NA	~2h	~56m	~3m	~16s	~32s	NA
8VQV	NA	~3s	~6m	~27m	~1h	NA	~35m	~24m	~3m	NA	~6s	NA
8VVJ	~3m	~2s	~5m	~27m	~38m	NA	~36m	~24m	NA	NA	~6s	NA
9BZ1	~4m	~5s	~4m	~28m	~39m	NA	~6h	~38m	NA	~6s	~14s	NA
9BZC	~5m	~5s	~5m	~29m	~56m	NA	~10h	~24m	NA	~6s	~16s	NA
9C75	NA	~7s	~3m	~36m	~32m	~27m	~41m	~35m	NA	~6s	~9s	~14m
9CBU	NA	~3m	NA	~5h	~2h	NA	~2d	~5h	NA	~3m	~9m	NA
9DCF	NA	~7s	~4m	~30m	~1h	~23m	NA	NA	~2m	~11s	~11s	NA
9ELY	NA	~41s	~16m	~3h	~55m	NA	~1d	~2h	NA	~0s	~2m	NA
9J6Y	NA	~2m	NA	NA	~2h	NA	NA	~22h	NA	~10m	~22m	NA
9ISV	NA	~2m	NA	NA	NA	NA	NA	NA	NA	NA	NA	NA
9J3R	NA	~12m	NA	NA	NA	NA	NA	NA	NA	NA	NA	NA
